# Integrative analysis of the prognostic value and immune microenvironment of mitophagy-related signature for multiple myeloma

**DOI:** 10.1186/s12885-023-11371-7

**Published:** 2023-09-12

**Authors:** Yachun Jia, Rui Liu, Luyi Shi, Yuandong Feng, Linlin Zhang, Ni Guo, Aili He, Guangyao Kong

**Affiliations:** 1 National and Local Joint Engineering Research Center of Biodiagnosis and Biotherapy, The Second Affiliated Hospital of Xi’an Jiaotong University, Xi’an, Shaanxi PR China; 2 Department of Hematology, The Second Affiliated Hospital of Xi’an Jiaotong University, Xi’an, Shaanxi PR China; 3 Precision Medical Institute, The Second Affiliated Hospital of Xi’an Jiaotong University, Xi’an, Shaanxi PR China; 4 Key Laboratory of Environment and Genes Related to Diseases, Xi’an Jiaotong University, Xi’an, Shaanxi PR China

**Keywords:** Mitophagy, Multiple myeloma, Risk signature, Nomogram, Immune infiltration, Prognosis

## Abstract

**Background:**

Multiple myeloma (MM) is a fatal malignant tumor in hematology. Mitophagy plays vital roles in the pathogenesis and drug sensitivity of MM.

**Methods:**

We acquired transcriptomic expression data and clinical index of MM patients from NCI public database, and 36 genes involved in mitophagy from the gene set enrichment analysis (GSEA) database. Least absolute shrinkage and selection operator (LASSO) Cox regression analysis was conducted to construct a risk score prognostic model. Kaplan–Meier survival analysis and receiver operation characteristic curves (ROC) were conducted to identify the efficiency of prognosis and diagnosis. ESTIMATE algorithm and immune-related single-sample gene set enrichment analysis (ssGSEA) was performed to uncover the level of immune infiltration. QRT-PCR was performed to verify gene expression in clinical samples of MM patients. The sensitivity to chemotherapy drugs was evaluated upon the database of the genomics of drug sensitivity in cancer (GDSC).

**Results:**

Fifty mitophagy-related genes were differently expressed in two independent cohorts. Ten out of these genes were identified to be related to MM overall survival (OS) rate. A prognostic risk signature model was built upon on these genes: VDAC1, PINK1, VPS13C, ATG13, and HUWE1, which predicted the survival of MM accurately and stably both in training and validation cohorts. MM patients suffered more adverse prognosis showed more higher risk core. In addition, the risk score was considered as an independent prognostic element for OS of MM patients by multivariate cox regression analysis. Functional pathway enrichment analysis of differentially expressed genes (DEGs) based on risk score showed terms of cell cycle, immune response, mTOR pathway, and MYC targets were obviously enriched. Furthermore, MM patients with higher risk score were observed lower immune scores and lower immune infiltration levels. The results of qRT-PCR verified VDAC1, PINK1, and HUWE1 were dysregulated in new diagnosed MM patients. Finally, further analysis indicated MM patients showed more susceptive to bortezomib, lenalidomide and rapamycin in high-risk group.

**Conclusion:**

Our research provided a neoteric prognostic model of MM based on mitophagy genes. The immune infiltration level based on risk score paved a better understanding of the participation of mitophagy in MM.

**Supplementary Information:**

The online version contains supplementary material available at 10.1186/s12885-023-11371-7.

## Highlights

The study reported a mitophagy-related genes signature in multiple myeloma.

The mitophagy-related genes signature had ideal prognostic independence in multiple myeloma.

A nomogram to predict the overall survival of multiple myeloma was built by combining the five-gene signature, LDH and ISS stage.

Immune infiltration was related to the mitophagy-related risk signature.

**Supplementary Information:**

The online version contains supplementary material available at 10.1186/s12885-023-11371-7.

## Introduction

Multiple myeloma (MM) is a fatal hematologic cancer featured with abnormal propagation of monoclonal plasma cells in bone marrow [1]. Over the past decades, many effective treatments, such as bortezomib (injectable proteasome inhibitor), lenalidomide (oral immunomodulatory drug), chimeric antigen receptor-engineered T cells, and autologous stem cell transplantation [2–5], improve the outcome of MM patients. However, the medical need of MM remains unmet as a result of the significant heterogeneity [6]. Various factors are involved in MM progress, mainly including genetic abnormalities [7], changes in bone marrow microenvironment (BM-ME) [8], and epigenetic alterations [9]. Studies confirmed that immune cells in BM-ME and the dysregulation of genes involved in immune checkpoint are related to the immune infiltration level of BM-ME [10, 11]. The protection from BM-ME and high genetic instability guard MM cells against chemotherapies, or receptor-targeting drugs, which virtually leads to resistance and relapse [12].

Mitochondria is not only a crucial house of producing energy via oxidative phosphorylation, but also a center of producing cellular metabolites [13]. Mitophagy is a process of lysosome-dependent mitochondrial autophagy, which protects cells against proapoptotic proteins, poisonous reactive oxygen species (ROS), and the unavailing hydrolytic action of adenosine triphosphate (ATP), induced by the depolarization of mitochondrial membrane or the mitochondrial DNA (mtDNA) changes [14, 15]. Accumulating evidences have recently confirmed that mitophagy is a double-edged sword in cancer development. On one hand, the decrease of mitophagy promotes the cancer progression [16]. On the other hand, the increased mitophagy facilitates cancer cells proliferation and progression by defending cancer cells from apoptosis [17]. Nonetheless, roles of mitochondrial dysfunction in the immune microenvironment and prediction of outcome in MM remain indistinct.

In our study, we performed the least absolute shrinkage and selection operator (LASSO) Cox regression analysis to build a five-mitophagy-related-gene prognostic risk signature model. The risk model revealed a great predicted value of actual survival probabilities. In addition, enrichment analysis identified the alteration of immune checkpoint and immune microenvironment based on the risk score. Finally, drug sensitivity analysis predicted latent drugs for treating MM.

## Materials and methods

### Data acquisition

MM transcriptomic expression data and clinical features were acquired from the NCI Gene Expression Omnibus database (GEO). The raw data was normalized and log2 transformed. All detailed information of GEO dataset were shown in Supplementary Table 1. The overall design of this study was shown in Supplementary Fig. 1. Among them, GSE6477 and GSE13591 were applied to appraisal differentially expressed genes (DEGs). GSE9782 was used as the training set to establish the prognostic risk score model, and GSE24080 and GSE4204 were utilized as validation cohorts. GSE24080 was applied to perform univariate analysis and multivariate analysis for overall survival (OS) rate and construct the nomogram model. GSE6477 and GSE47552 were applied to evaluate the performance of risk score for MM diagnosis.

#### Establishment of the prognostic risk model

First, univariate cox regression analysis was applied to obtain the OS-related genes with p < 0.05, and ten genes (SLC25A4, VDAC1, RNF41, SLC25A5, PINK1, SQSTM1, VPS13C, ATG13, HUWE1, and OPTN) were significantly correlated with MM OS time. Next, we performed LASSO Cox regression to optimize the prognostic model by further compressing the genes and constructing the prognostic model by “glmnet” package (version 4.1-1), and five genes finally came into the risk score formula (VDAC1, PINK1, VPS13C, ATG13, and HUWE1). Moreover, MM patients were divided into two groups upon the optimal cutoff of the risk score with “Survminer” package (version 0.4.9). Receiver operating characteristic (ROC) curve was applied to estimate the prognostic value of risk score model in MM patients.

#### Construction of the nomogram

To assess the prediction value of risk score in MM, we performed univariate and multivariate cox regression analysis. Variables with p < 0.10 was supposed to the multivariate cox regression analysis, and p < 0.05 was considered as remarkable independent prognostic factors. Then, we used “rms” package (version 6.2-0) to construct the nomogram independent prognostic model. Finally, we assessed the predictive value of nomogram by ROC curve and calibration curve.

#### Functional pathway enrichment analysis

We used the online website Metascape (http://metascape.org/gp/index.html) to analyze gene ontology (GO) and Kyoto Encyclopedia of Genes and Genomes (KEGG) pathway [18–20] of DEGs based on risk score group. We executed Gene set enrichment analysis (GSEA) by GSEA software (version 4.1.0). The absolute value of NES value > 1, p value < 0.05, and false discovery rate (FDR) value < 0.25 showed significance.

#### Estimation of immune infiltration

We computed the immune score, stromal score, tumor purity, and ESTIMATE score for each sample in GSE24080 by ESTIMATE algorithm [21]. According to the previous study [22], single sample GSEA (ssGSEA) was conducted to evaluate the abundance of infiltrating cells by “GSVA” package (version 1.38.2).

#### Prediction of drug sensitivity

To achieve precise treatment upon mitophagy-related signature and identified potential drugs for MM, we utilized the Genomics of drugs sensitivity in cancer (GDSC, https://www.cancerrxgene.org/) to predict the chemotherapeutic response [23]. R package “pRROphetic” was implemented to evaluate the half-maximal inhibitory concentration (IC_50_) by ridge regression [24]. The forecast precision was determined through 10-fold cross-validation using the GDSC training set. Additional methods are described in supplementary methods.

### Statistical analysis

At least three dependent experiments were performed in qRT-PCR and values were presented as mean ± SD. We used unpaired Student’s t-test and Mann-Whitney U test to judge the otherness in two groups. More than two groups, we used One-way ANOVA (for parametric data) and Kruskal-Wallis (for non-parametric data) test to contrast significance. ROC curve was performed to assess the diagnosis value of risk score and the prognostic value of nomogram model in MM. The Kaplan-Meier method with a two-sided log-rank test was performed to evaluate the OS of MM patients. SPSS 21 software (SPSS, Chicago, USA) and GraphPad Prism 8 were used for statistical analysis. P value lower than 0.05 was defined markedly different.

## Results

### Identification of 15 mitophagy-related genes differentially expressed in MM

**Fig. 1:**
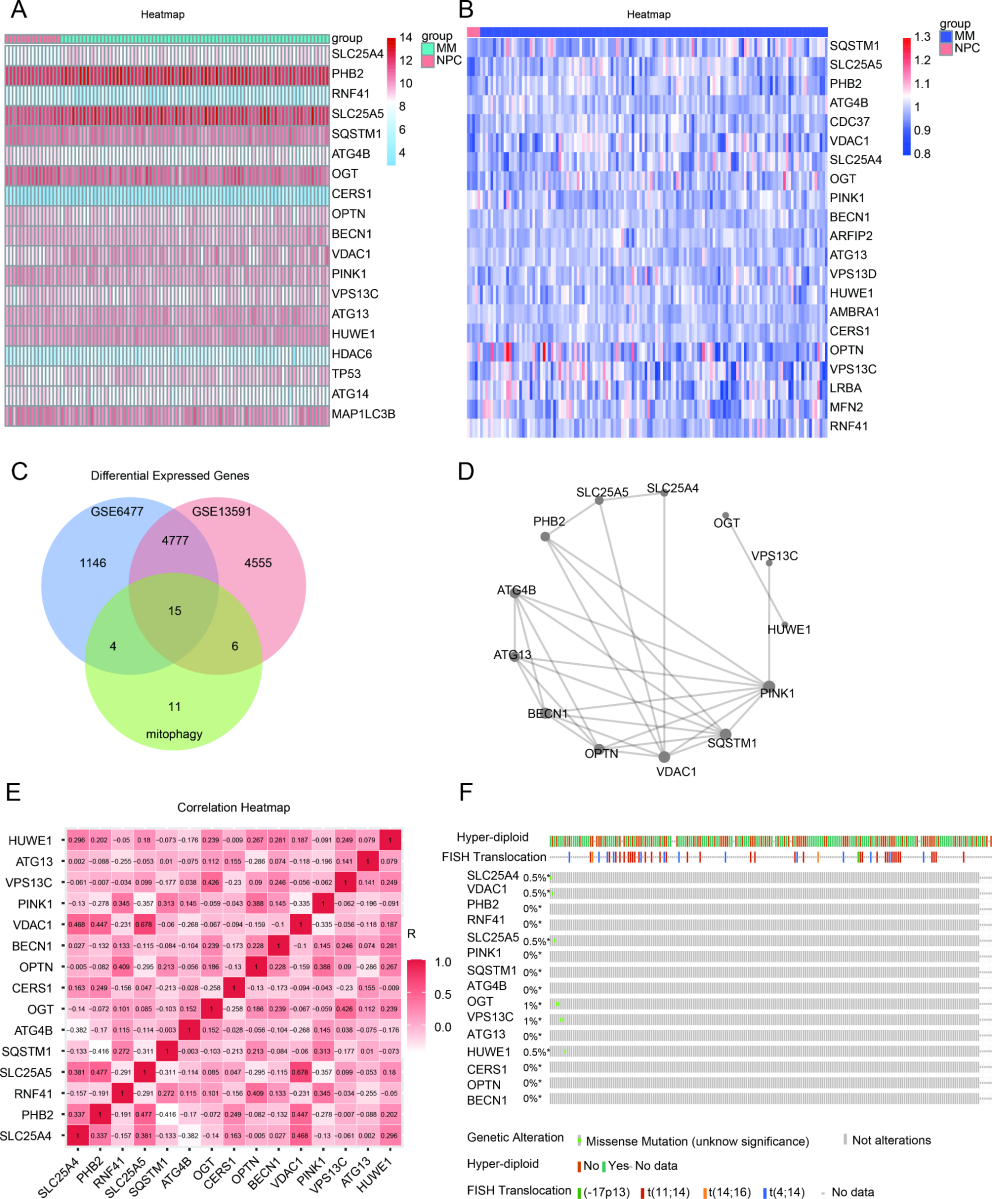
Identification of 15 mitophagy-related genes differentially expressed in MM. (**A**). 19 mitophagy-related genes were differentially expressed in GSE6477. (**B**). 21 mitophagy-related genes were differentially expressed in GSE13591. (**C**). The veen plot of overlapped mitophagy-related genes. (**D**). The PPI network of the 15 mitophagy-related genes. (**E**). The correlation of 15 mitophagy-related genes in GSE6477. (**F**). The mutant frequency of 15 mitophagy-related genes in 211 multiple myeloma samples, including chromosome translocation and hyper-diploid

We acquired a total of 36 mitophagy-related genes from GSEA GOBP database (supplementary Table 2). The datasets of GSE13591 (including 5 normal plasma cells (NPC) and 133 MM patients) and GSE6477 (including 15 NPC and 73 MM patients) were used to analyze the genes involved in mitophagy which were differentially expressed in MM. We found 19 genes and 21 genes were differentially expressed in MM patients compared to human healthy donors in GSE6477 and GSE13591, respectively (Fig. 1A and B). We observed that SLC25A4, PHB2, CERS1, VPS13C, HUWE1, VDAC1, and SLC25A5 were significantly upregulated, while OGT, ATG13, PINK1, and OPTN were significantly downregulated in MM patients (supplementary Fig. 2A and 2B). Then, venn plot was used to identify 15 mitophagy-related genes overlapped in the two datasets (Fig. 1C). To ulteriorly probe the relationship among 15 mitophagy-related genes, we performed PPI network analysis (Fig. 1D). We found PINK1, SQSTM1 and VDAC1 were hub genes. Besides, we conducted correlation analysis to ascertain the relationship among these genes (Fig. 1E). The correlation between VDAC1 and SLC25A5 was significantly positive (r = 0.678), whereas the correlation between SQSTM1 and PHB2 was significantly negative (r = -0.416). Furthermore, we used cBioPortal, an online database, and found no frequent mutation of 15 mitophagy-related genes in 211 MM samples (Fig. 1F) [25].

### Establishment of prognostic risk model upon on mitophagy-related genes

**Fig. 2:**
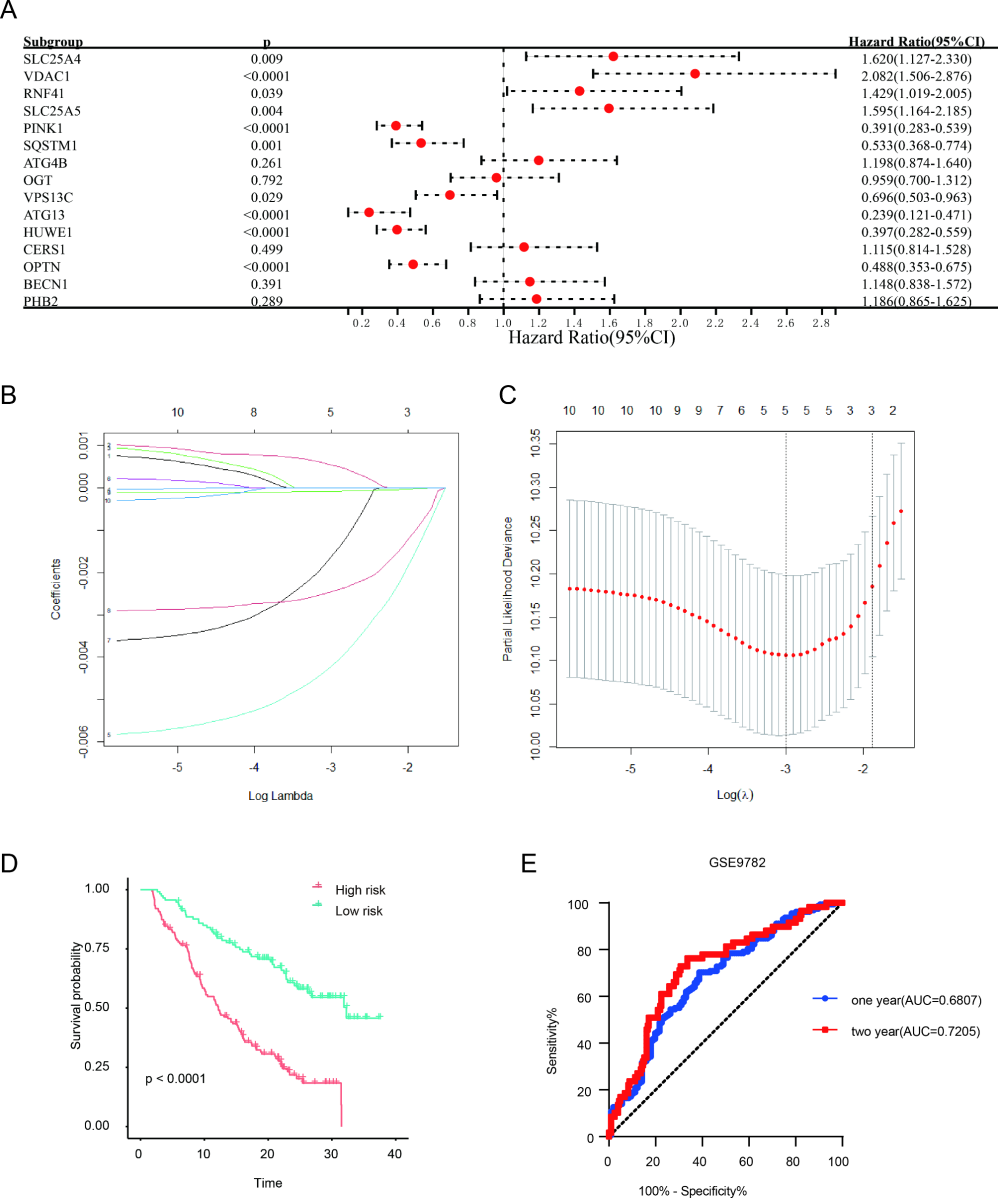
Construction of prognostic risk model based on mitophagy-related genes. (**A**). Forest plot of the univariate cox regression in GSE9782. (**B**). The LASSO Cox analysis identified mitophagy-related genes in the training cohort. (**C**). Partial likelihood deviance of different numbers of variables. One-thousand- fold cross-validation was applied for tuning penalty parameter selection. (**D**). Kaplan-Meier survival analysis in training cohort based on the risk score. (**E**). ROC curve for the prognostic risk signature. LASSO: least absolute shrinkage and selection operator; ROC: receiver operating characteristic

We chose GSE9782, which includes 264 MM patients with survival time, to screen the OS-related mitophagy-related genes. We found the low expression of ATG13 (p < 0.0001), HUWE1 (p < 0.0001), OPTN (p < 0.0001), PINK1 (p < 0.0001), SQSTM1 (p = 0.001) and VPS13C (p = 0.029) was notably related to adverse OS rate, while the high expression of SLC25A5 (p = 0.004), VDAC1 (p < 0.0001), RNF41 (p = 0.039), and SLC25A4 (p = 0.009) was notably related to poor OS (Fig. 2A). Then, we executed LASSO cox regression analysis to establish a prognostic model in the training cohort GSE9782 (Fig. 2B C). Five mitophagy-related genes, including VDAC1, PINK1, VPS13C, ATG13 and HUWE1, were involved in the risk model. We evaluated the total risk score of every patient with formula as follows: (5.476831e-04*the expression of VDAC1) + (-4.209033e-03* the expression of PINK1) + (-1.629760e-03* the expression of VPS13C) + (-2.450343e-03* the expression of ATG13) + (-7.849962e-05* the expression of HUWE1). On the bias of the cutoff risk score, 264 MM patients were separated into low- and high-risk group. Kaplan-Meier survival analysis found that MM patients had poorer outcome observably in high-risk group than that in low-risk score (p < 0.0001) (Fig. 2D). ROC curve suggested that the AUC values were 0.6807 (95% confidence interval (CI): 0.6147 to 0.7467, p < 0.0001) and 0.7205 (95%CI: 0.6471 to 0.7940, p < 0.0001) for survival rate of 1- year and 2-year, respectively (Fig. 2E). Next, the total risk score was calculated in other two independent cohorts for validation. MM patients had poorer OS with high-risk score than those with low-risk score in the GSE24080 (p = 0.0002) and GSE4204 (p < 0.0001) (supplementary Fig. 3A). The AUC values were 0.6236 (95%CI: 0.5064 to 0.7408, p = 0.0484), 0.6432 (95%CI: 0.5589 to 0.7275, p = 0.0028), and 0.6085 (95%CI: 0.5355 to 0.6015, p = 0.0038) for survival rate of 1-, 2-, and 3-year in GSE24080, respectively (supplementary Fig. 3B). Parallel values were 0.5739 (95%CI: 0.5180 to 0.6297, p < 0.0001), 0.5894 (95%CI: 0.5416 to 0.6373, p = 0.0003), and 0.6195 (95%CI: 0.5680 to 0.6710, p < 0.0001) for survival rate of 1-, 2- and 3-year in GSE4204, respectively (supplementary Fig. 3B).

### The mitophagy-related risk score was an independent prognostic element in MM

**Fig. 3:**
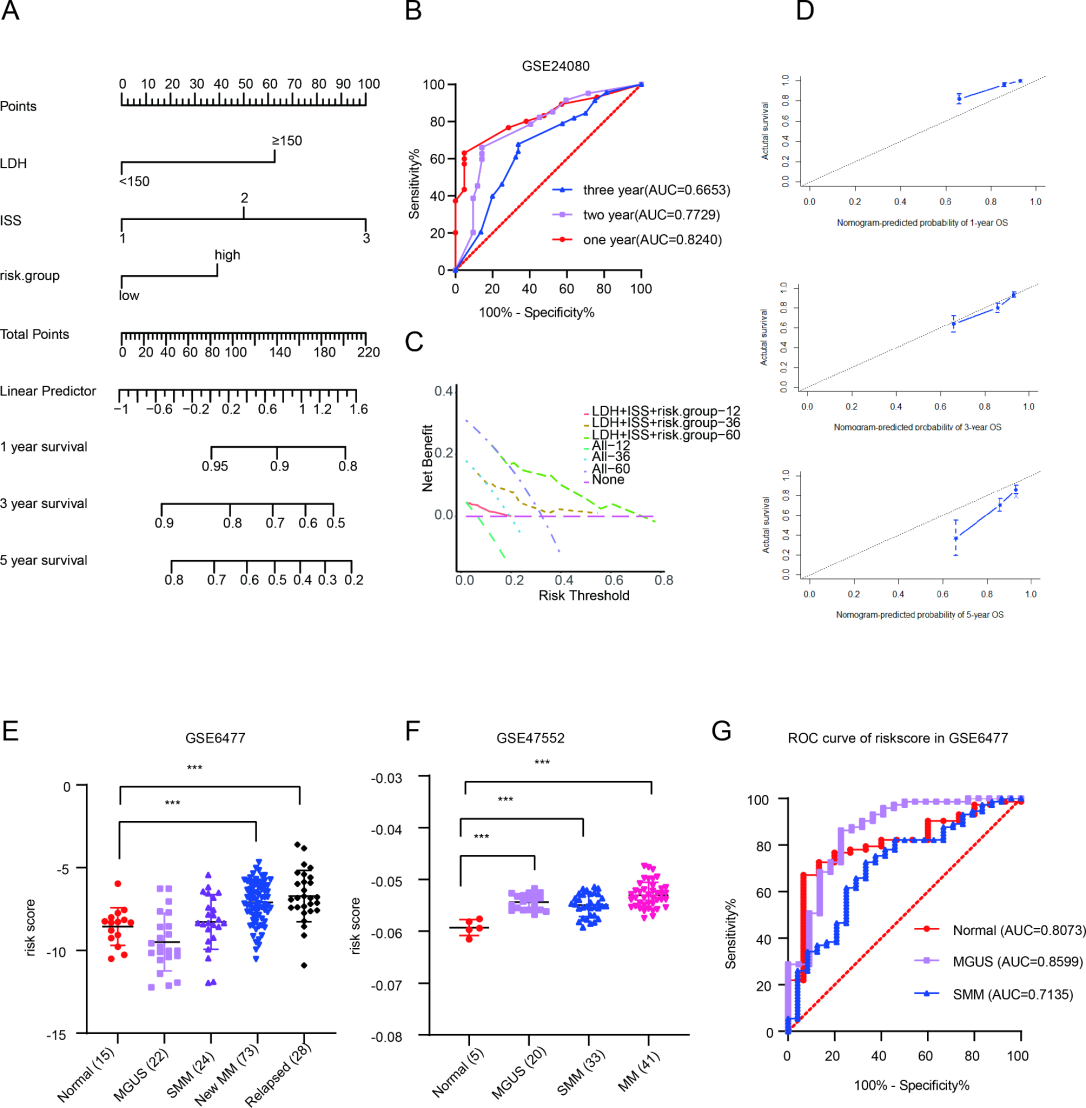
The mitophagy-related risk score was an independent prognostic factor in MM. (**A**). Nomogram predicting 1-, 3-, and 5-year survival for MM patients based on mitophagy-related genes risk score. To use this nomogram, the specific point for each variable of the patient lies on each variable axis. Draw a vertical line upward to determine the point at which each variable accepts; the sum of these points is located on the Total Points axis, and draw a vertical line down to the survival axis to determine the probability of 1-, 3- and 5- year overall survival. LDH: lactate dehydrogenase; ISS: international staging system. (**B**). ROC curve for the nomogram prognosis system. (**C**). DCA curve for the nomogram prognosis system. The x-axis represents the threshold probability, while the y-axis stands for the net benefits. (**D**). Calibration plot of the nomogram for 1-year, 3-year, and 5-year OS. The risk score in normal donors and MM patients with different stages in GSE6477 (**E**) and GSE47552 (**F**). (**G**). ROC curve for MM with different myeloma stages. DCA: decision curve analysis * *p* < 0.05, ** *p* < 0.01, *** *p* < 0.001

We applied GSE24080 database to performed univariate and multivariate cox regression analysis. We observed lactate dehydrogenase (LDH) (hazard ratio (HR), 2.134; 95% CI, 1.39–3.277; p = 0.001), international staging system (ISS) stage (HR, 1.83; 95% CI, 1.414–2.369; p < 0.0001), and risk score (HR, 1.601; 95% CI, 1.059–2.422; p = 0.026) were the independent prognostic elements for MM survival (Table 1). In order to visualize the prognosis prediction, we further constructed a nomogram model with the above independent prognostic features (Fig. 3A). Calibration curves are typically used to assess the agreement between predicted probabilities and observed event rates or frequencies in the real world, with the 45° line representing the best prediction scenario. Calibration ROC and curves were applied to estimate the precision and efficacy of the prognostic risk model. Decision curve analysis (DCA) is usually used to evaluate the clinical utility of different predictive models [26]. We observed that the precision of the total points in nomogram for 1- year survival was 0.8240 (95%CI: 0.7570 to 0.8911, P < 0.0001), 2- year survival 0.7729 (95%CI: 0.6912 to 0.8545, P < 0.0001), and 3- year survival 0.6653 (95%CI: 0.5933 to 0.7372, P < 0.0001), respectively (Fig. 3B). The calibration curves and DCA revealed a great predicted value of actual survival probabilities for 1-year, 3-year, and 5-year, respectively base on the nomogram model (Fig. 3C and D).

**Table 1:** Univariate analysis and Multivariate analysis for OS in GSE24080

variables	univariate analysis		multivariate analysis				
	HR (95%CI)	p value	beta	SE	Wald	HR (95%CI)	*p* value
Age, years	0.953(0.593–1.531)	0.842					
Gender	1.103 (0.733–1.660)	0.638					
Race	1.145(0.639–2.052)	0.649					
LDH(U/l)	2.564 (1.685–3.903)	**< 0.0001**	0.758	0.219	12.021	2.134 (1.39–3.277)	**0.001**
ALB(g/l)	0.533(0.339–0.838)	**0.006**	-0.181	0.246	0.537	0.835 (0.515–1.353)	0.464
HGB(g/dl)	0.546(0.310–0.961)	**0.036**	0.163	0.314	0.268	1.177 (0.636–2.179)	0.605
ISS stage	1.984(1.582–2.487)	**< 0.0001**	0.604	0.132	21.046	1.83 (1.414–2.369)	**< 0.0001**
Risk score	1.823(1.212–2.744)	**0.004**	0.471	0.211	4.976	1.601(1.059–2.422)	**0.026**

Bold values indicate statistically significant *p* values less than 0.05

The development of MM includes 4 steps: monoclonal gammopathy of unknown significance (MGUS), smoldering multiple myeloma (SMM), multiple myeloma, and plasma cell leukemia (PCL) or refractory and/or relapse MM (RRMM). The results found that MM patients and RRMM patients had more dramatically higher risk score than that in normal people (Fig. 3E F). Besides, ROC curve suggested that the value of risk score was able to distinguish MM from healthy donor (AUC = 0.8073, 95%CI: 0.6962 to 0.9184, *P* = 0.0002), MGUS (AUC = 0.8599, 95%CI: 0.7622 to 0.9516, P < 0.0001), and SMM (AUC = 0.7135, 95%CI: 0.5939 to 0.8330, *P* = 0.0018) (Fig. 3G).

### The mitophagy-related risk signature was related to clinical characteristics of MM

Next, we used the database of GSE24080 to estimate the relation between the risk score and clinical characteristics (Table 2). The results showed that the value of risk score was related to LDH (p = 0.03), albumin (ALB) (p = 0.025), hemoglobin (HGB) (p = 0.02), beta-2 microglobulin (B2M) (p = 0.037), ISS stage (p = 0.022), the percentage of plasma cells in bone marrow biopsy (BMPC) (p = 0.018), and cytogenetic abnormalities (p < 0.0001). Cytogenetic abnormalities has been regarded as a poor prognostic factor for MM [27]. Therefore, we choose GSE136337 as the validation dataset, which consists with several cytogenetic information. We observed that MM patients with cytogenetic abnormalities, including del13q (p < 0.0001), del1p32 (p < 0.0001), del1p (p < 0.0001), del1q (p < 0.0001), myc_8q24 (p = 0.001), x1qplus (p < 0.0001), and hyperdiploid (p < 0.0001) had higher risk score (Table 3). But we don’t find the relationship between risk score and t (4;14), t (14;20), and t (14;16), because the number limitation of MM patients with these cytogenetic abnormalities. Further, we analyzed the gene levels of five mitophagy-related genes in the risk score formula. We observed MM patients with cytogenetic abnormalities had higher VDAC1 expression and lower PINK1 expression (supplementary Table 3). These results indicated mitophagy-related risk signature was associated with clinical characteristics of MM, especially cytogenetic abnormalities.

**Table 2:** The correlation of prognostic risk signature and clinical characteristics

Clinical characteristics	Number	Risk score, mean ± SD	*P* value
**Gender**			0.265
male	195	-0.0765 ± 0.00019	
female	118	-0.0769 ± 0.00023	
**Age**			
< 65	236	-0.0767 ± 0.00016	0.959
≥ 65	77	-0.0767 ± 0.00032	
**Race**			0.290
White	270	-0.0766 ± 0.00016	
others	43	-0.0771 ± 0.00038	
**LDH(U/L)**			
< 150	156	-0.0770 ± 0.00019	**0.03**
≥ 150	157	-0.0763 ± 0.00022	
**ALB(g/L)**			
< 3.5	57	-0.0760 ± 0.00037	**0.025**
≥ 3.5	256	-0.0768 ± 0.00016	
**HGB(g/Dl)**			
< 9	26	-0.0755 ± 0.00068	**0.02**
≥ 9	287	-0.0768 ± 0.00015	
**B2M(mg/l)**			**0.037**
< 2.9 ≥ 2.9	158 155	-0.0770 ± 0.00019 -0.0764 ± 0.00022	
**ISS stage**			
I	194	-0.0768 ± 0.00017	**0.022**
II	59	-0.0770 ± 0.00038	
III	60	-0.0759 ± 0.00034	
**BMPC, %**			**0.018**
< 46	157	-0.0771 ± 0.00020	
≥ 46	156	-0.0762 ± 0.00021	
**Cytogenetic abnormalities**			**< 0.0001**
Yes	117	-0.0759 ± 0.00024	
No	196	-0.0771 ± 0.00018	
**Bone lesions**			
Yes No	210 103	-0.0765 ± 0.00018 -0.0769 ± 0.00025	0.243

46% was the media value of BMPC

The bold values indicate statistically different (*p* < 0.05)

**Table 3:** Impact of risk-score on the most common cytogenetic abnormalities of multiple myeloma (GSE136337)

Cytogenetic abnormalities	Number	Risk score, mean ± SD	*P* value
**del13q**			**< 0.0001**
False	349	-0.0574 ± 0.0026	
Ture	77	-0.0561 ± 0.0025	
**del11q**			
False	418	-0.0572 ± 0.0026	0.273
Ture	8	-0.0562 ± 0.0019	
**del17p**			0.181
False	422	-0.0572 ± 0.0026	
Ture	4	-0.0555 ± 0.0023	
**del16q**			
False	412	-0.0572 ± 0.0026	0.093
Ture	14	-0.0560 ± 0.0024	
**del1p32**			
False	341	-0.0574 ± 0.0026	**< 0.0001**
Ture	85	-0.0527 ± 0.0026	
**del1p**			
False	336	-0.0574 ± 0.0026	**< 0.0001**
Ture	90	-0.0563 ± 0.0026	
**del1q**			**< 0.0001**
False Ture	339 87	-0.0574 ± 0.0026 -0.0563 ± 0.0026	
**amp1q**			
False	422	-0.0572 ± 0.0026	0.314
Ture	4	-0.0559 ± 0.0039	
**myc_8q24**			**0.001**
False	406	-0.0573 ± 0.0026	
Ture	20	-0.0554 ± 0.0025	
**x1qplus**			**< 0.0001**
False	324	-0.0575 ± 0.0026	
Ture	102	-0.0562 ± 0.0024	
**T (11,14)**			
False Ture	404 22	-0.0572 ± 0.0026 -0.0574 ± 0.0030	0.704
**hyperdiploid**			**< 0.0001**
False	341	-0.0575 ± 0.0026	
Ture	85	-0.0561 ± 0.0024	

Bold values indicate statistically significant *p* values less than 0.05

**Fig. 4:**
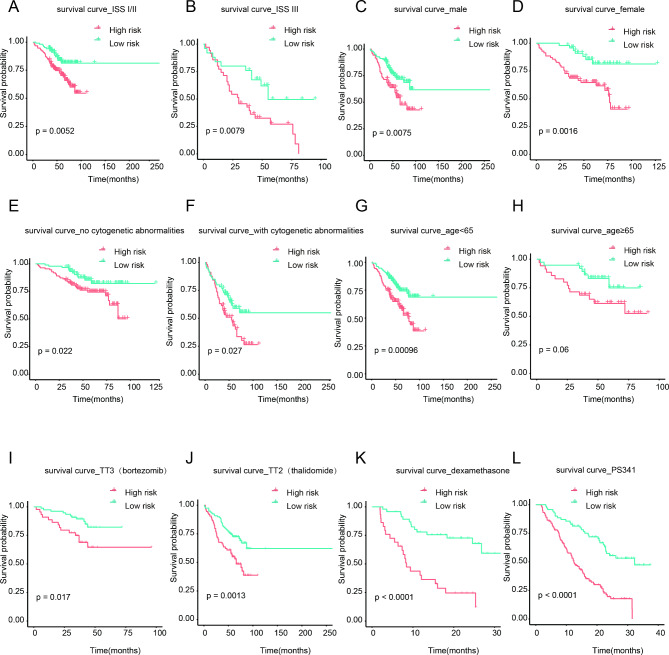
Survival analysis in different subgroup. Kaplan–Meier survival analysis of MM patients grouped by ISS stage I/II/III (**A, B**), male and female (**C, D**), no cytogenetic abnormalities and with cytogenetic abnormalities (**E, F**), age < 65 and ≥ 65 (**G, H**), bortezomib treatment and thalidomide treatment (**I, J**) in GSE24080. Kaplan–Meier survival analysis of MM patients stratified by dexamethasone treatment and PS341 treatment (**K, L**) in GSE9782.

To further assess the applicability of the risk signature model for different MM conditions, we performed Kaplan–Meier survival analysis. The risk score showed stabilized predictive value in term of ISS stage (ISS I/II, p = 0.0052, ISS III, p = 0.0079, Fig. 4A and B), gender (male, p = 0.0075; female, p = 0.0016, Fig. 4C and D), the condition of cytogenetic (no cytogenetic abnormalities, p = 0.022, with cytogenetic abnormalities, p = 0.027, Fig. 4E F). Among these, MM patients had the shorter OS in higher-risk score group. However, this phenomenon was found in young people (p = 0.00096, Fig. 4G), but not in older people (p = 0.06, Fig. 4H). In addition, higher risk score was related to more adverse OS in MM patients received various treatment, including bortezomib (p = 0.017, Fig. 4I), thalidomide (p = 0.0013, Fig. 4J), dexamethasone (DEX, p < 0.0001, Fig. 4K), and PS341 (proteasome inhibitor, p < 0.0001, Fig. 4L). Taken together, the prognostic model satisfied the personalized medical needs of MM.

### Gene expression and functional pathway enrichment analysis of mitophagy-related risk signature in MM

**Fig. 5:**
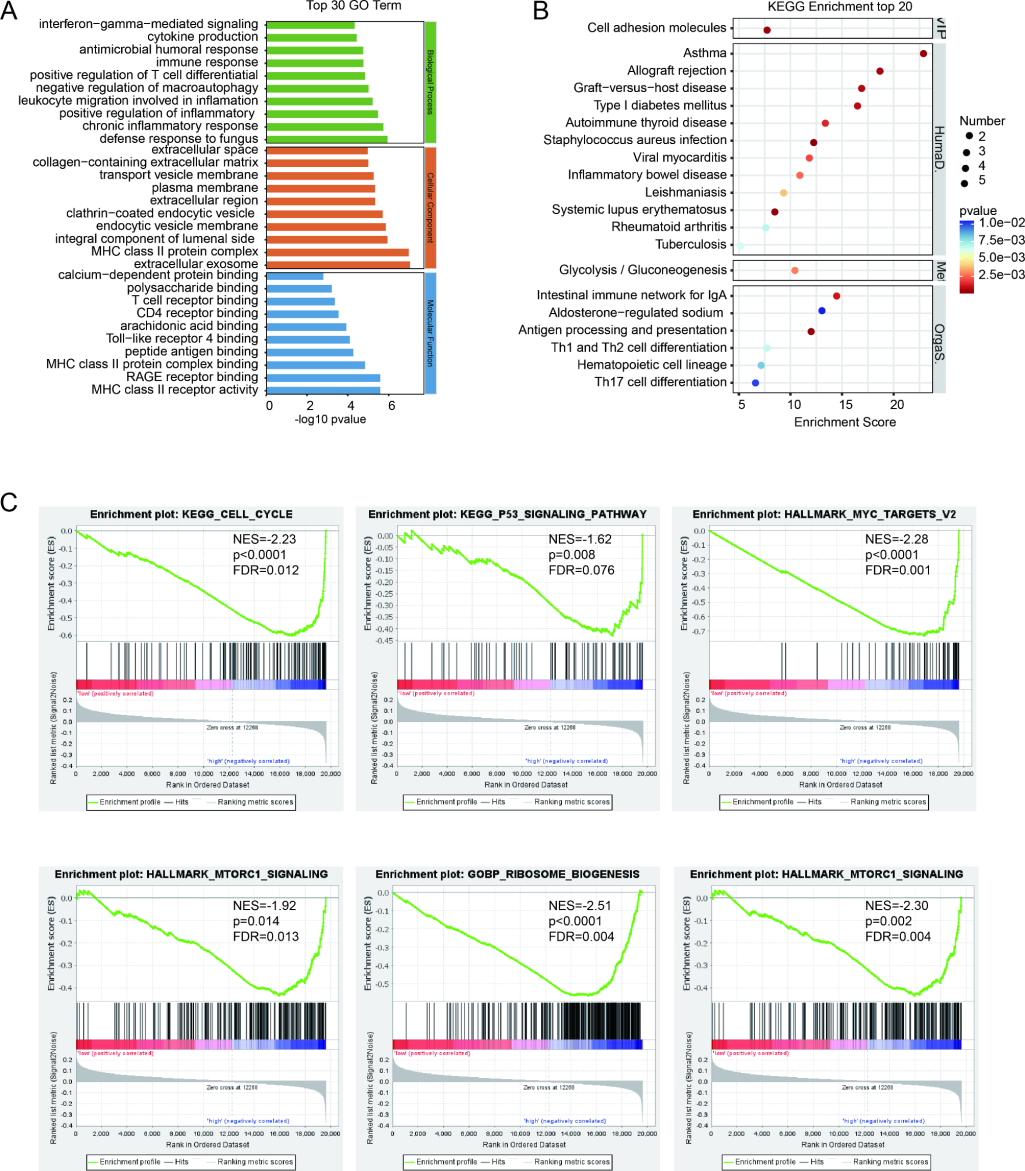
Enrichment analysis of mitophagy-related risk signature. Gene ontology and KEGG pathway enrichment analysis of DEGs in GSE24080 dataset grouped by risk score. (**A**). The significantly enriched gene ontology biological process (GOBP) of downregulated DEGs. (**B**). The significantly enriched KEGG pathway of downregulated DEGs. (**C**). GSEA analysis results of KEGG gene set, HALLMARK gene set, and GOBP gene set. NES, normalized enrichment score; FDR, false discovery rate

To further explore the underlying mechanisms of mitophagy-related genes in MM, we used limma package to get DEGs in GSE24080 based on the risk score. A total of 83 increased genes and 80 decreased genes were discerned (supplementary Fig. 4A and supplementary Table 4). Enrichment analysis of DEGs uncovered that upregulated genes were markedly enriched in mitotic cell cycle, ATPase activity, and ATP binding in GO term (supplementary Fig. 4B), and p53 signaling pathway, cell cycle, Hippo signaling pathway, FoxO signaling pathway, and TGF-beta signaling pathway in KEGG (supplementary Fig. 4C). Downregulated genes were overtly enriched in term of immune response, cytokine production, T cell receptor binding, and Toll-like receptor binding in GO term (Fig. 5A), and Hematopoietic cell lineage, cell adhesion molecules, and Th17 cell differentiation in KEGG (Fig. 5B). Moreover, GSEA revealed that high-risk group significantly enriched tumorigenesis and mitochondrial terms, including cell cycle (NES=-2.23, p < 0.0001, FDR = 0.012), p53 signaling pathway (NES=-1.62, p = 0.008, FDR = 0.076) in KEGG gene set, MYC targets (NES=-2.48, p < 0.0001, FDR = 0.0005), G2M checkpoint (NES=-2.25, p < 0.0001, FDR = 0.001), E2F target (NES=-2.17, p < 0.0001, FDR = 0.003), and mTORC1 signaling (NES=-1.92, p = 0.014, FDR = 0.013) in HALLMARK gene set, and ribosome biogenesis (NES=-2.51, p < 0.0001, FDR = 0.004), and mitochondrial gene expression (NES=-2.30, p = 0.002, FDR = 0.004) in GOBO gene set (Fig. 5C and supplementary Fig. 4D).

### Immune infiltration was related to the mitophagy-related risk signature

**Fig. 6:**
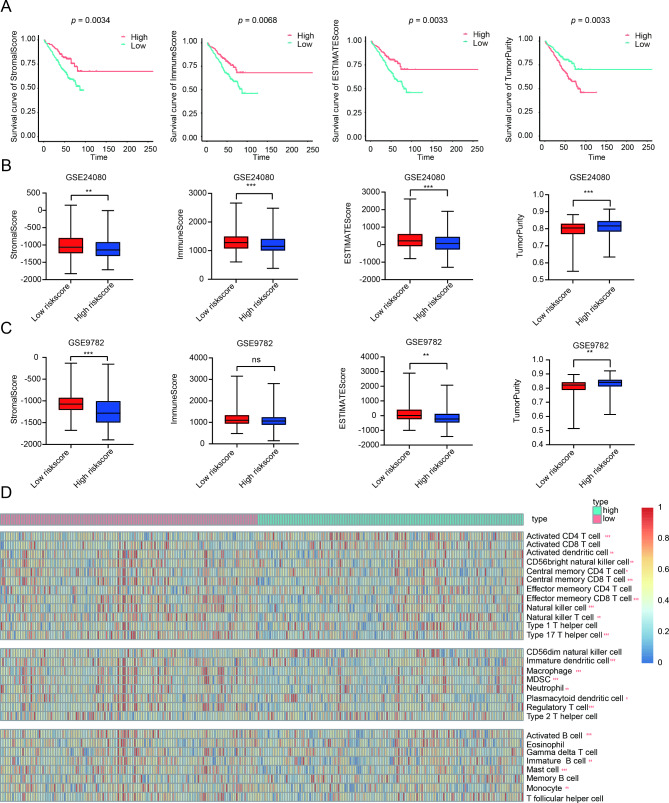
Immune infiltration was related to the mitophagy-related risk signature. (**A**). Kaplan-Meier survival analysis upon stromal score, immune score, ESTIMATE score, and tumor purity. (**B**). The distribution of stromal score, immune score, ESTIMATE score, and tumor purity upon risk score in GSE24080. (**C**). The distribution of stromal score, immune score, ESTIMATE score, and tumor purity upon risk score in GSE9782. (**D**). The heatmap of the comparison in 28 immune-related gene sets upon risk score in GSE24080. * *p* < 0.05, ** *p* < 0.01, *** *p* < 0.001

Due to the significantly enrichment of immune response in downregulated DEGs in MM patients, ESTIMATE was performed to assess the different immune infiltration levels of MM patients in different risk group. First, Kaplan-Meier analysis revealed that low stromal score, immune score and ESTIMATE score were associated with adverse OS, while high tumor purity was associated with poor OS (Fig. 6A). In addition, MM patients had lower levels of stromal score, immune score and ESTIMATE score in the high-risk group, and higher levels of tumor purity in both training cohort and validation cohort (Fig. 6B C). Furthermore, we conducted ssGSEA to probe the disparate immune cell subsets based on the risk score. The results found 19 of 28 immune cells were notably dysregulated in high-risk group (Fig. 6D). Activated B cell, central memory CD4 T cell, activated dendritic cell, central memory CD8 T cell, CD56 bright killer cell, effector memory CD8 T cell, immature B cell, nature killer cell, nature killer T cell, and type 17 T helper cell, participated in anti-tumor immune response, were all decreased in high-risk group. Besides, the levels of immature dendritic cell, regulatory T cell, MDSC, macrophage and mast cell, belonging to immunosuppressive cells, were also decreased in MM patients with high-risk score.

**Fig. 7:**
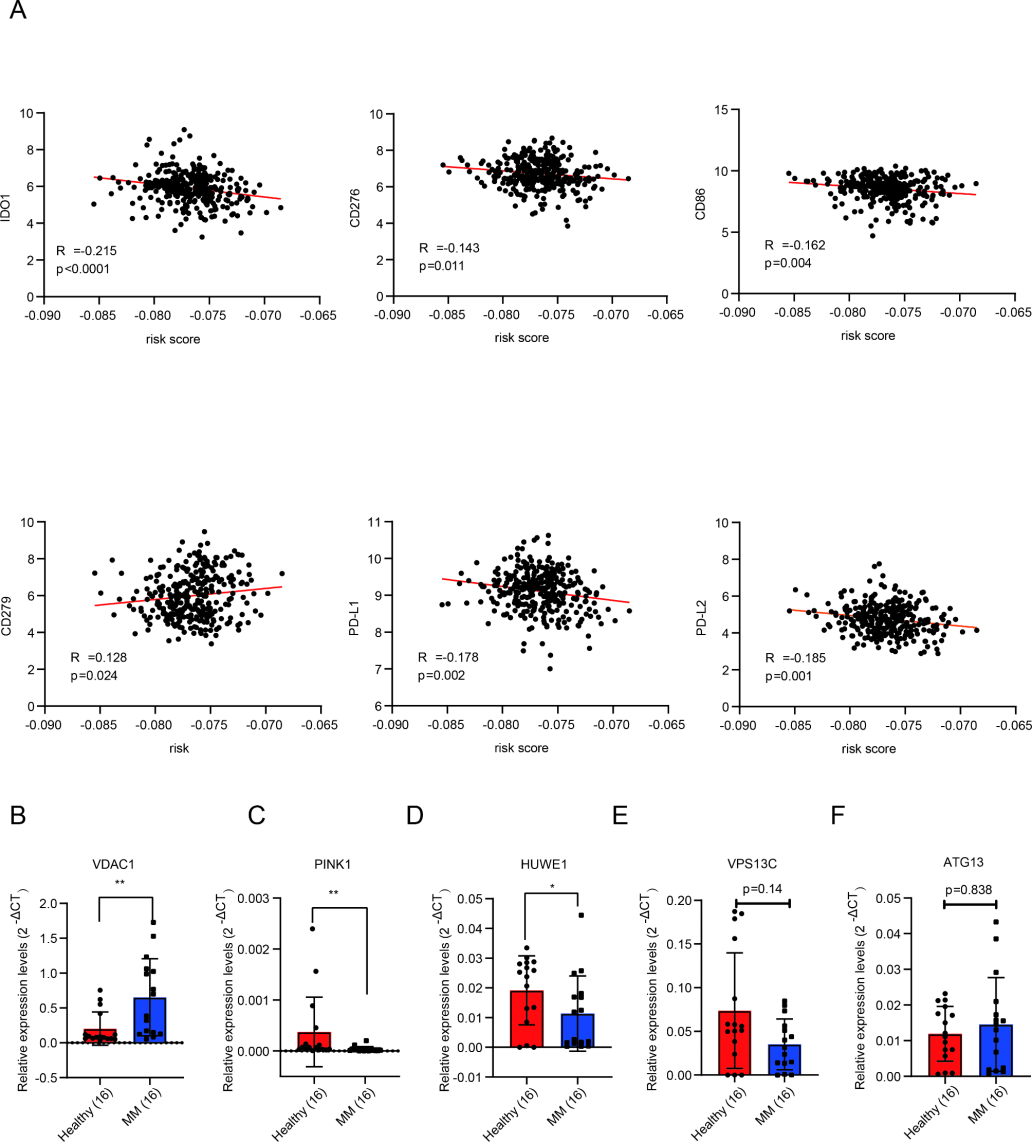
Validations for mitophagy-related genes in clinical MM samples. (**A**). Correlation analysis between risk score and immune checkpoint for tumor-targeted treatment. The mRNA expression of VDAC1 (**B**), PINK1 (**C**), HUWE1 (**D**), VPS13C (**E**), and ATG13 (**F**) by qRT-PCR in primary bone marrow mononuclear cells from NDMM and normal donors. Data represent the mean ± SD. * *p* < 0.05, ** *p* < 0.01, *** *p* < 0.001

Moreover, we explored the relation between risk score and immune checkpoint, which was regarded as potential therapy targets for MM [28]. Intriguingly, the results showed that risk score was connected with immune checkpoint negatively, including IDO1, CD276, CD86, PD-L1, and PD-L2, while positively related to CD279 (Fig. 7A). Taken together, the prognostic risk score was connected with tumor immune infiltration levels and genes involved in immune checkpoint. Therefore, our research verified the immune checkpoint inhibitors, including PD-L1, IDO1, CD276, CD86 had potential clinical values for MM patients.

### Validation for mitophagy-related genes in MM samples

To validate these results of bioinformatic analysis, we collected the mononuclear cells of NDMM patients and healthy donors in bone marrow. Then, the qRT-PCR was executed. We observed the expression levels of VDAC1 were markedly upregulated in MM patients than that in healthy donors (P = 0.0056, Fig. 7B), while the expression levels of PINK1 and HUWE1 were downregulated in MM patients (P = 0.001, P = 0.019, Fig. 7C and D). Although, the expression level of VPS13C was lower and ATG13 was higher in MM patients than these in controls, no evident differences were observed (P = 0.14, P = 0.838, Fig. 7E F), maybe for the reason of insufficient sample size.

### Prediction of possible drugs for MM upon risk score signature

**Fig. 8:**
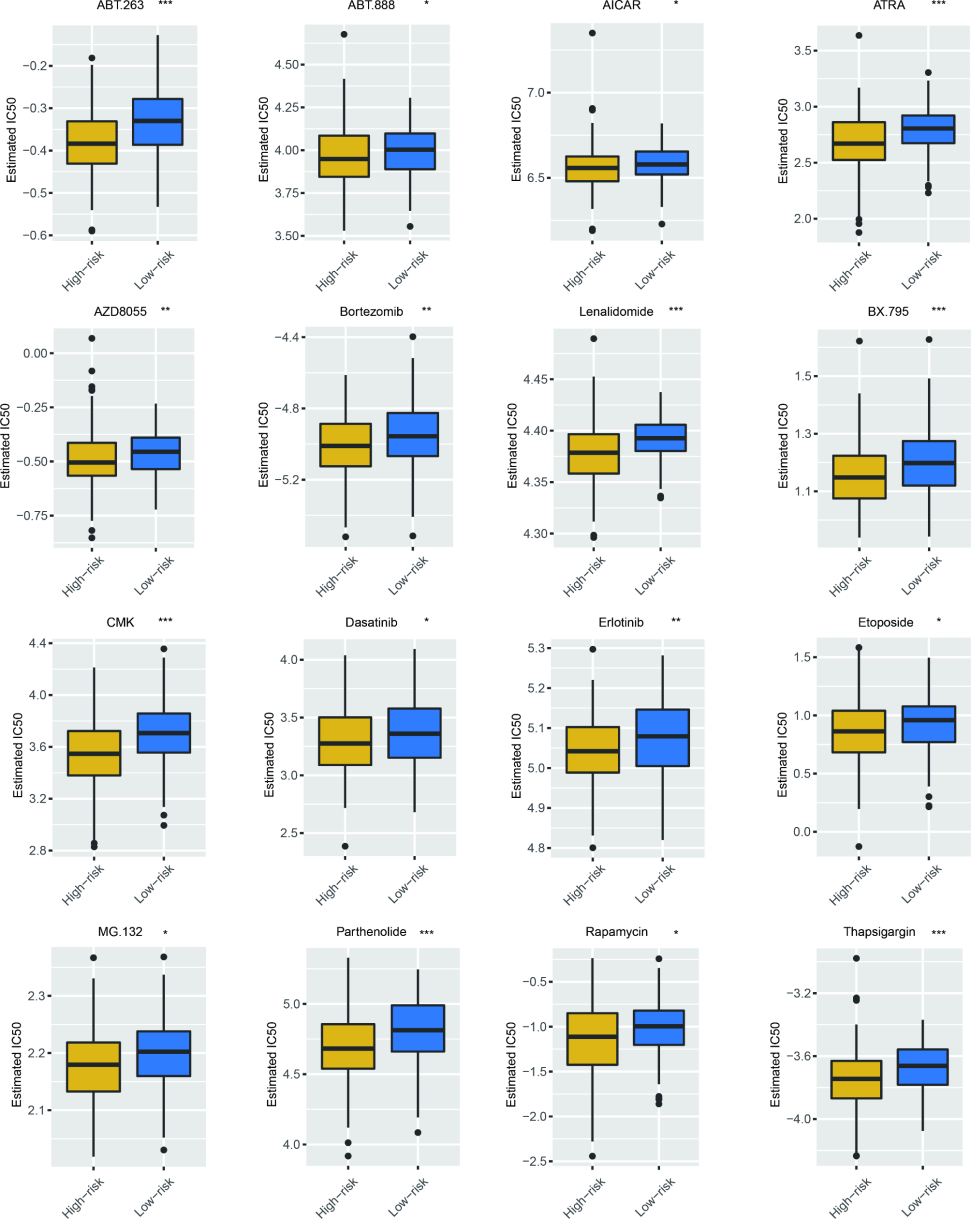
Prediction of potential drugs for MM based on risk score signature. The distribution of the IC_50_ of various drugs upon risk score in GSE24080.

To further identified potential drugs for MM, we explored the estimated half-maximal inhibitory concentration (IC_50_) between the two group on the bias of the Genomics of Drug Sensitivity in Cancer (GDSC). We found decreased IC_50_ of 16 drugs in MM patients with high-risk score, including bortezomib and lenalidomide, which are widely used in MM therapy (Fig. 8). Besides, the estimated IC_50_ of ABT.263 (bcl-2 inhibitor), ABT. 888 (PARP inhibitor), AICAR (AMPK activator), ATRA (all-trans retinoic acid), dasatinib (tyrosine kinase inhibitor), AZD8055 (mTOR inhibitor), erlotinib (EGFR inhibitor), etoposide, MG.132 (proteasome inhibitor), parthenolide (HDAC inhibitor), rapamycin (mTORC1 complex inhibitor), and thapsigargin (ATPase inhibitor) were also lower in high-risk score group (Fig. 8), which might provide novel insights into MM treatment. To sum up, the risk score can be considered as an index to choose appreciate therapeutic targets for MM precisely.

## Discussion

As the second most common hematological malignancy, MM remains incurable because of drug resistance and relapse [29]. There is no excellent survival prediction for MM so far. Mitophagy is a vital cellular progress, which results in degradation of dysfunctional mitochondria. Several proteins have been reported involved in mitophagy, including PINK1, Parkin, OPTN, ATG13, and p62/SQSTM1, and FUNDC1, etc. [30]. Up to now, increasing researches have revealed that mitophagy acts essential part in the development and drug sensitivity of various cancers, especially in MM. It has been reported that Parkin/PARK2 carried mutation in glioma [31], lung cancer [32], and breast cancer [33]. In addition, the expression of thioredoxin was notably increased in MM cells, which resistant to bortezomib. Increased thioredoxin increased resistance to bortezomib in MM via mitophagy inactivation [34]. However, as far as we known, there is no research focus on the potential relationship between mitophagy-related genes and MM prognosis.

Herein, we constructed a five-mitophagy-related risk signature for MM. The prognostic model worked well in both training and validation cohort via ROC curve and Kaplan-Meier survival analysis. Further enrichment analysis displayed that the DEGs between high- and low-risk groups were observably enriched in term of cell cycle, ATP-binding, and tumor-related pathways. Immune infiltration analysis had manifested that MM patients in high-risk score group suffered a notably decreased condition of antitumor immune activity. Finally, drug sensitivity analysis provided potential strategies for treating MM.

In our study, the mitophagy-related risk signature consists of VDAC1, PINK1, VPS13C, ATG13, and HUWE1, which can accurately predict MM prognosis. MM patients in high-risk score group suffered more adverse OS. Our findings are consistent with previous studies. VDAC1(voltage-dependent anion channel 1), a main factor in the outer mitochondrial membrane, was upregulated in multiple cancers, including lung cancer [35], breast tumor [36], cervical tumor [37], and liver cancer [38]. Overexpressed VDAC1 was a prognostic element and was related to immune infiltrates in breast cancer [36]. Besides, VDAC1 involved in bromodomain inhibitor resistance in breast cancer [39]. VDAC1 was upregulated in mesothelioma patients. The expression levels of VDAC1 were positive related to disease stage and high expression of VDAC1 had correlation with shorter survival rates. Loss of VDAC1 could inhibit cell propagation in mesothelioma cancer cells [40]. PINK1 (PTEN-induced kinase 1)/Parkin was a classical axis to regulate mitophagy in mammalian cells [41]. Previous researches uncovered that PINK1 and Parkin decreased in colorectal cancer [42]. Loss of PINK1 restrained mitophagy, facilitated the Warburg effect, and promoted macrophages polarization in gastric cancer [43]. Besides, another study found PINK1 was reduced in MM and regulated the MOB1B-mediated Hippo‐YAP/TAZ pathway leading to MM migration and homing [44]. Matrine, a natural alkaloid, accelerated the apoptosis of liver cancer cells by intercepting the PINK1/Parkin pathways and restraining mitophagy [45]. Consistent with our research, Li et al. identified an autophagy-related signature for MM prognosis, which contained PINK1, EIF2AK2, KIF5B, MYC, NRG2, and VEGFA [46]. These finding revealed the important role of PINK1 in MM. Recent study revealed that VPS13C (vacuolar protein sorting 13 homolog C) was descended and associated with poor prognosis in skin cutaneous melanoma [47]. ATG13 (autophagy related 13) is a member of autophagy initiation complex. ULK1 could active autophagy via phosphorylating ATG13 to inhibit the progress of breast cancer [48]. Zhou et al. reported that estrogen receptor α (ERα) could bind the promoter of ATG13 leading to the increasing of mitophagy. Erα inhibitor oxabicycloheptene sulfonate could reduce the expression of ATG13, leading to restrain the viability of breast cancer cells [49]. Niu et al. reveled that chemotherapy drug licochalcone A could active the upstream of autophagy ULK1/ATG13 complex, inducing hepatocellular carcinoma cells apoptosis by inducing autophagy [50]. Previous studied demonstrated HUWE1 played disparate role in different tumors, as an oncogene in liver cancer, lung cancer, colon adenocarcinoma, and stomach adenocarcinoma, whereas an antioncogene in skin tumors, and thyroid cancer [51]. The reason for the opposite function of HUWE1 in tumor may be the different tumor microenvironment and genetic backgrounds. The function and mechanism of these five mitophagy-related genes in various tumors had been overwhelmingly illustrated. However, the further study is urgently needed to be conducted in MM.

GSEA results indicated oncogene-related gene set, including MYC targets, E2F target, and mTORC1 signaling was significantly associated with high-risk group. Our findings were in line with the lower rapamycin IC_50_ in the high-risk group by subsequently drug sensitivity analysis, revealing the potential role of mTOR inhibitors in MM therapy. In fact, there is a cross-talk between mTOR signaling pathway and ubiquitin proteasome system [52]. The combination of these drugs offers multiple possibilities for treating MM. A clinical trial aims to RRMM (NCT00483262) uncovered the synergistic effect of bortezomib and mTOR inhibitor. In this clinical trial, 14 of 43 MM patients obtained partial response or better [53].

The results of ESTIMATE and ssGSEA disclosed that MM patients with high-risk score suffered lower levels of stromal score, immune score and ESTIMATE score, whereas higher levels of tumor purity. Besides, cell populations participated in anti-tumor immune response, such as, immature B cell, central memory CD4 T cell, type 17 T helper cell, central memory CD8 T cell, CD56 bright killer cell, effector memory CD8 T cell, nature killer cell, and nature killer T cell, were all markedly reduced in high-risk score group. Research has confirmed the essentials of mitochondrial dynamics in immune cells [54]. Paul et al. revealed the elevation of mitophagy facilitated anti-tumor immunity in intestinal epithelial cells by activated CD8 T cell through cross-dressing of dendritic cells [55]. Moreover, the activation of anti-tumor immune cells demands energy. The lessen antitumor activity of immune cells may be associated with mitophagy dysfunction [56]. In addition, the levels of immature dendritic cell, regulatory T cell, MDSC, macrophage and mast cell, belong to immunosuppressive cells, were also decreased in high-risk score group. A study revealed that macrophages was as a harmful prognostic index in innate immunity [57]. To sum up, the results indicated the tight correlation between mitophagy-related risk score and immunosuppression, which may explain the reason of poor prognosis in MM patients with high risk.

New strategies for the treatment of tumors focus on immune checkpoint inhibitors. We ulteriorly probed the correlation between risk score and immune checkpoint. These results disclosed that risk score had negative association with immune checkpoint, including IDO1, CD276, CD86, PD-L1, and PD-L2, which indicated inhibitors targeting checkpoints, such as PD-L1 may be less valid in MM patients in high-risk score group. However, a clinical trial has been reported that RRMM patients with extramedullary disease obtained benefit from the combination of PD-L1 inhibitor avelumab with radiotherapy [58]. Therefore, these findings suggested that mitophagy status and sensibility to immune checkpoints may be heterogeneous between primary and recurrent MM patients.

However, there are still some limitations in our study. First, the expression of five-mitophagy-genes were verified only in mRNA level, and the expression at protein level should be further clarified. Second, the nomogram model was not be applied in validation cohort, due to the lack of clinical information in the two independent datasets. Third, the function of the five-mitophagy-genes in MM has not yet to be cleared. Therefore, further experiments *in vitro and in vivo* need to be conducted.

## Conclusions

In conclusion, we constructed a five-mitophagy-genes (VDAC1, PINK1, VPS13C, ATG13, and HUWE1) prognostic risk model, which as an independent element for MM OS, could estimate the survival of MM accurately and stably both in training and validation cohorts. The molecular landscape characteristics upon the risk score, including the regulatory pathways, immune infiltration level, and potential drug targets, improved our cognition for MM, which provide novel insights into MM treatment.

### Electronic supplementary material

Below is the link to the electronic supplementary material.

Supplementary Material 1

Supplementary Material 2

Supplementary Material 3

## Data Availability

The public datasets used in this article, including GSE6477, GSE13591, GSE9782, GSE24080, GSE4204, and GSE47552 can be downloaded in the GEO database. The original contributions presented in the study are included in the article/ Supplementary Material. Further inquiries can be directed to the corresponding authors.

## Abbreviations

MM: Multiple myeloma
BM-ME: Bone marrow microenvironment
ROS: Reactive oxygen species
ATP: Adenosine triphosphate
mtDNA: Mitochondrial DNA
LASSO: Least absolute shrinkage and selection operator
GEO: Gene Expression Omnibus database
DEGs: Differentially expressed genes
S: Overall survival
PPI: Protein-Protein interaction
ROC: Receiver operating characteristic
GO: Gene ontology
KEGG: Kyoto Encyclopedia of Genes and Genomes
GSEA: Gene set enrichment analysis
FDR: False discovery rate
ssGSEA: Single sample GSEA
GDSC: Genomics of drugs sensitivity
IC_50_: Half-maximal inhibitory concentration
qRT-PCR: Real-time polymerase chain reaction
BMMC: Bone marrow mononuclear cells
LDH: Lactate dehydrogenase
HR: Hazard ratio
CI: Confidence interval
MGUS: Monoclonal gammopathy of unknown significance
SMM: Smoldering multiple myeloma
RRMM: Refractory and/or relapse MM
ALB: Albumin
HGB: Hemoglobin
B2M: Beta-2 microglobulin
VDAC1: Voltage-dependent anion channel 1
PINK1: PTEN-induced kinase 1
VPS13C: Vacuolar protein sorting 13 homolog C
ATG13: Autophagy related 13
PD-L1: Programmed cell death-ligand 1
PD-L2: Programmed cell death-ligand 2

## References

[1] Kumar SK, Rajkumar V, Kyle RA, Duin MV, Anderson KC (2017). Multiple myeloma. Nat Rev Dis Primers.

[2] Natural history of. Relapsed myeloma, refractory to immunomodulatory drugs and proteasome inhibitors: a multicenter IMWG study. Leukemia 2017. 10.1038/leu.2017.138 28620163

[3] Rajkumar SV, Kumar S. Multiple myeloma current treatment algorithms. Blood Cancer J 2020, 10(9). 10.1038/s41408-020-00359-2 PMC7523011 32989217

[4] Zhao WH, Wang BY, Chen LJ, Fu WJ, Xu J, Liu J, Jin SW, Chen YX, Cao XM, Yang Y et al. Four-year follow-up of LCAR-B38M in relapsed or refractory multiple myeloma: a phase 1, single-arm, open-label, multicenter study in China (LEGEND-2). J Hematol Oncol 2022, 15(1). 10.1186/s13045-022-01301-8 PMC9261106 35794616

[5] Pinto V, Bergantim R, Caires HR, Seca H, Guimaraes JE, Vasconcelos MH. Multiple myeloma: available therapies and causes of Drug Resistance. Cancers 2020, 12(2). 10.3390/cancers12020407 PMC7072128 32050631

[6] Abdallah N, Rajkumar SV, Greipp P, Kapoor P, Gertz MA, Dispenzieri A, Baughn LB, Lacy MQ, Hayman SR, Buadi FK et al. Cytogenetic abnormalities in multiple myeloma: association with disease characteristics and treatment response. *Blood Cancer J* 2020, 10(8). 10.1038/s41408-020-00348-5 PMC7419564 32782240

[7] Corre J, Munshi N, Avet-Loiseau H. Genetics of multiple myeloma: another heterogeneity level? *Blood* 2015, 125(12):1870–6. 10.1182/blood-2014-10-567370 PMC4366624 25628468

[8] Bianchi G, Munshi NC (2015). Pathogenesis beyond the cancer clone(s) in multiple myeloma. Blood.

[9] De Smedt E, Maes K, Verhulst S, Lui H, Kassambara A, Maes A, Robert N, Heirman C, Cakana A, Hose D (2018). Loss of RASSF4 expression in multiple myeloma promotes RAS-Driven malignant progression. Cancer Res.

[10] Guillerey C, Harjunpaa H, Carrie N, Kassem S, Teo T, Miles K, Krumeich S, Weulersse M, Cuisinier M, Stannard K (2018). TIGIT immune checkpoint blockade restores CD8(+) T-cell immunity against multiple myeloma. Blood.

[11] Di Marzo L, Desantis V, Solimando AG, Ruggieri S, Annese T, Nico B, Fumarulo R, Vacca A, Frassanito MA (2016). Microenvironment drug resistance in multiple myeloma: emerging new players. Oncotarget.

[12] Holthof LC, Mutis T. Challenges for Immunotherapy in multiple myeloma: bone marrow microenvironment-mediated Immune suppression and Immune Resistance. Cancers 2020, 12(4). 10.3390/cancers12040988 PMC7226482 32316450

[13] Richardson DR, Lane DJ, Becker EM, Huang ML, Whitnall M, Suryo Rahmanto Y, Sheftel AD, Ponka P (2010). Mitochondrial iron trafficking and the integration of iron metabolism between the mitochondrion and cytosol. Proc Natl Acad Sci U S A.

[14] Lemasters JJ (2014). Variants of mitochondrial autophagy: types 1 and 2 mitophagy and micromitophagy (type 3). Redox Biol.

[15] Chang JY, Yi HS, Kim HW, Shong M (2017). Dysregulation of mitophagy in carcinogenesis and tumor progression. Bba-Bioenergetics.

[16] Yu MF, Nguyen ND, Huang YQ, Lin D, Fujimoto TN, Molkentine JM, Deorukhkar A, Kang Y, San Lucas FA, Fernandes CJ et al. Mitochondrial fusion exploits a therapeutic vulnerability of pancreatic cancer. Jci Insight 2019, 4(16). 10.1172/jci.insight.126915 PMC6777817 31335325

[17] Maes H, Rubio N, Garg AD, Agostinis P (2013). Autophagy: shaping the tumor microenvironment and therapeutic response. Trends Mol Med.

[18] Kanehisa M, Furumichi M, Sato Y, Kawashima M, Ishiguro-Watanabe M (2023). KEGG for taxonomy-based analysis of pathways and genomes. Nucleic Acids Res.

[19] Kanehisa M (2019). Toward understanding the origin and evolution of cellular organisms. Protein Sci.

[20] Kanehisa M, Goto S (2000). KEGG: Kyoto Encyclopedia of genes and genomes. Nucleic Acids Res.

[21] Yoshihara K, Shahmoradgoli M, Martínez E, Vegesna R, Kim H, Torres-Garcia W, Treviño V, Shen H, Laird PW, Levine DA (2013). Inferring tumour purity and stromal and immune cell admixture from expression data. Nat Commun.

[22] Jia QZ, Wu W, Wang YQ, Alexander PB, Sun CD, Gong ZH, Cheng JN, Sun HB, Guan YF, Xia XF et al. Local mutational diversity drives intratumoral immune heterogeneity in non-small cell lung cancer. Nat Commun 2018, 9. 10.1038/s41467-018-07767-w PMC6299138 30560866

[23] Lu XF, Jiang LY, Mang LY, Zhu Y, Hu WJ, Wang JS, Ruan XJ, Xu ZB, Meng XW, Gao J (2019). Immune signature-based subtypes of cervical squamous cell Carcinoma tightly Associated with Human Papillomavirus Type 16 expression, molecular features, and clinical outcome. Neoplasia.

[24] Geeleher P, Cox N, Huang RS. pRRophetic: an R package for prediction of clinical chemotherapeutic response from tumor gene expression levels. (1932–6203 (Electronic)). 10.1371/journal.pone.0107468 PMC4167990 25229481

[25] Cerami E, Gao J, Dogrusoz U, Gross BE, Sumer SO, Aksoy BA, Jacobsen A, Byrne CJ, Heuer ML, Larsson E (2012). The cBio cancer genomics portal: an open platform for exploring multidimensional cancer genomics data. Cancer Discov.

[26] Fitzgerald M, Saville BR, Lewis RJ (2015). Decision curve analysis. Jama-J Am Med Assoc.

[27] Morgan GJ, Walker BA, Davies FE (2012). The genetic architecture of multiple myeloma. Nat Rev Cancer.

[28] Zanwar S, Nandakumar B, Kumar S. Immune-based therapies in the management of multiple myeloma. Blood Cancer J 2020, 10(8). 10.1038/s41408-020-00350-x PMC7443188 32829378

[29] Robak P, Drozdz I, Szemraj J, Robak T (2018). Drug resistance in multiple myeloma. Cancer Treat Rev.

[30] Mizushima N, Levine B (2010). Autophagy in mammalian development and differentiation. Nat Cell Biol.

[31] Maugeri G, D’Amico AG, Magro G, Salvatorelli L, Barbagallo GMV, Saccone S, Drago F, Cavallaro S (2015). D’Agata V: expression profile of parkin isoforms in human gliomas. Int J Oncol.

[32] D’Amico AG, Maugeri G, Magro G, Salvatorelli L, Drago F, D’Agata V (2015). Expression pattern of parkin isoforms in lung adenocarcinomas. Tumor Biol.

[33] Tay SP, Yeo CWS, Chai C, Chua PJ, Tan HM, Ang AXY, Yip DLH, Sung JX, Tan PH, Bay BH (2010). Parkin enhances the expression of cyclin-dependent kinase 6 and negatively regulates the proliferation of breast Cancer cells. J Biol Chem.

[34] Zheng ZH, Fan SJ, Zheng J, Huang W, Gasparetto C, Chao NJ, Hu JD, Kang YB. Inhibition of thioredoxin activates mitophagy and overcomes adaptive bortezomib resistance in multiple myeloma. J Hematol Oncol 2018, 11. 10.1186/s13045-018-0575-7 PMC5828316 29482577

[35] Zhang GX, Jiang GX, Wang C, Zhong K, Zhang JJ, Xue Q, Li X, Jin H, Li BL (2016). Decreased expression of microRNA-320a promotes proliferation and invasion of non-small cell lung cancer cells by increasing VDAC1 expression. Oncotarget.

[36] Fang YT, Liu JP, Zhang QC, She CH, Zheng RJ, Zhang RD, Chen ZX, Chen CF, Wu JD. Overexpressed VDAC1 in breast cancer as a novel prognostic biomarker and correlates with immune infiltrates. World J Surg Oncol 2022, 20(1). 10.1186/s12957-022-02667-2 PMC9215028 35729567

[37] Zhang CL, Ding WC, Liu Y, Hu Z, Zhu D, Wang XL, Yu L, Wang LM, Shen H, Zhang WC (2016). Proteomics-based identification of VDAC1 as a tumor promoter in cervical carcinoma. Oncotarget.

[38] Pittala S, Krelin Y, Shoshan-Barmatz V (2018). Targeting Liver Cancer and Associated Pathologies in mice with a mitochondrial VDAC1-Based peptide. Neoplasia.

[39] Yang GC, Zhou DW, Li J, Wang W, Zhong W, Fan W, Yu MC, Cheng HT (2019). VDAC1 is regulated by BRD4 and contributes to JQ1 resistance in breast cancer. Oncol Lett.

[40] Pandey SK, Machlof-Cohen R, Santhanam M, Shteinfer-Kuzmine A, Shoshan-Barmatz V. Silencing VDAC1 to treat Mesothelioma Cancer: Tumor Reprograming and Altering Tumor Hallmarks. Biomolecules 2022, 12(7). 10.3390/biom12070895 PMC9312978 35883451

[41] Eiyama A, Okamoto K (2015). PINK1/Parkin-mediated mitophagy in mammalian cells. Curr Opin Cell Biol.

[42] Poulogiannis G, McIntyre RE, Dimitriadi M, Apps JR, Wilson CH, Ichimura K, Luo FJ, Cantley LC, Wyllie AH, Adams DJ (2010). PARK2 deletions occur frequently in sporadic colorectal cancer and accelerate adenoma development in apc mutant mice. P Natl Acad Sci USA.

[43] Xu Y, Lu JW, Tang YB, Xie WJ, Zhang HT, Wang BB, Zhang SL, Hou WJ, Zou C, Jiang PC (2022). PINK1 deficiency in gastric cancer compromises mitophagy, promotes the Warburg effect, and facilitates M2 polarization of macrophages (retracted article. See vol. 549, 2022). Cancer Lett.

[44] Fan SJ, Price T, Huang W, Plue M, Warren J, Sundaramoorthy P, Paul B, Feinberg D, MacIver N, Chao N et al. PINK1-Dependent Mitophagy regulates the Migration and Homing of multiple myeloma cells via the MOB1B-Mediated Hippo-YAP/TAZ pathway. Adv Sci 2020, 7(5). 10.1002/advs.201900860 PMC7055555 32154065

[45] Wei R, Cao J, Yao S. Matrine promotes liver cancer cell apoptosis by inhibiting mitophagy and PINK1/Parkin pathways. (1466 – 1268 (Electronic)). 10.1007/s12192-018-0937-7 PMC6237690 30209783

[46] Li LC, Chen T, Wang JS, Li MX, Li QS. Identification of an Autophagy-Related Signature Based on Whole Bone Marrow Sequencing for the Prognosis and Immune Microenvironment Characterization of Multiple Myeloma. *J Immunol Res* 2022, 2022. 10.1155/2022/3922739 PMC9169202 35677537

[47] Wu XP, Zhao JM. Novel oxidative stress-related prognostic biomarkers for melanoma associated with tumor metastasis. Medicine 2021, 100(8). 10.1097/MD.0000000000024866 PMC7909214 33663112

[48] Karabi Z, Moradian F, Kheirabadi M (2022). The effect of lactoferrin on ULK1 and ATG13 genes expression in breast cancer cell line MCF7 and bioinformatics studies of protein interaction between lactoferrin and the autophagy initiation complex. Cell Biochem Biophys.

[49] Zhou J, Shen R, Zhou HB, Huang J. OBHS impairs the viability of breast cancer via decreasing ERα and Atg13. (1090–2104 (Electronic)). 10.1016/j.bbrc.2021.08.013 34388457

[50] Niu Q, Zhao W, Wang J, Li CM, Yan T, Lv W, Wang GJ, Duan WH, Zhang T, Wang KN (2018). LicA induces autophagy through ULK1/Atg13 and ROS pathway in human hepatocellular carcinoma cells. Int J Mol Med.

[51] Gong XF, Du DY, Deng YR, Zhou YQ, Sun L, Yuan ST (2020). The structure and regulation of the E3 ubiquitin ligase HUWE1 and its biological functions in cancer. Invest New Drug.

[52] Eichner R, Fernandez-Saiz V, Targosz BS, Bassermann F (2019). Cross Talk Networks of mammalian target of Rapamycin Signaling with the Ubiquitin Proteasome System and their clinical implications in multiple myeloma. Int Rev Cel Mol Bio.

[53] Ghobrial IM, Weller E, Vij R, Munshi NC, Banwait R, Bagshaw M, Schlossman R, Leduc R, Chuma S, Kunsman J (2011). Weekly bortezomib in combination with temsirolimus in relapsed or relapsed and refractory multiple myeloma: a multicentre, phase 1/2, open-label, dose-escalation study. Lancet Oncol.

[54] Xie JH, Li YY, Jin J (2020). The essential functions of mitochondrial dynamics in immune cells. Cell Mol Immunol.

[55] Ziegler PK, Bollrath J, Pallangyo CK, Matsutani T, Canli O, De Oliveira T, Diamanti MA, Muller N, Gamrekelashvili J, Putoczki T (2018). Mitophagy in Intestinal epithelial cells triggers adaptive immunity during tumorigenesis. Cell.

[56] O’Sullivan TE, Johnson LR, Kang HH, Sun JC (2015). BNIP3-and BNIP3L-Mediated Mitophagy promotes the generation of natural killer cell memory. Immunity.

[57] Zhou SL, Zhou ZJ, Hu ZQ, Huang XW, Wang Z, Chen EB, Fan J, Cao Y, Dai Z, Zhou J (2016). Tumor-Associated Neutrophils Recruit Macrophages and T-Regulatory cells to promote progression of Hepatocellular Carcinoma and Resistance to Sorafenib. Gastroenterology.

[58] Kazandjian D, Dew A, Hill E, Ramirez EG, Morrison C, Mena E, Lindenberg L, Yuan C, Maric I, Wang HW (2021). Avelumab, a PD-L1 inhibitor, in combination with Hypofractionated Radiotherapy and the Abscopal Effect in Relapsed Refractory multiple myeloma. Oncologist.

