# Supplementary material for: Integrative analysis of the prognostic value and immune microenvironment of mitophagy-related signature for multiple myeloma
Source: BMC Cancer. 2023 Sep 12;23:859. doi: 10.1186/s12885-023-11371-7 (PMC10496355; doi:10.1186/s12885-023-11371-7)
Supplement: Supplementary file 1 — Supplementary Material 1 [file 12885_2023_11371_MOESM1_ESM.docx]

Supplementary Material


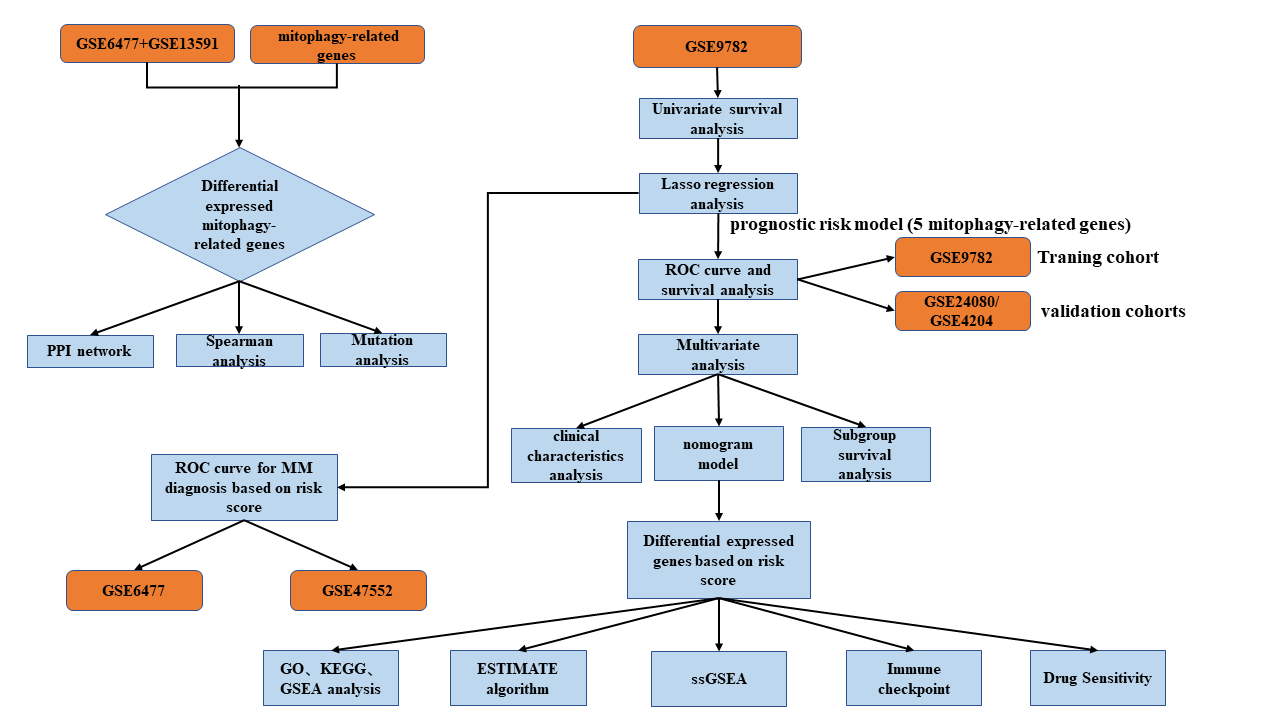


**Supplementary Figure 1 The overall design and workflow of this study**

**
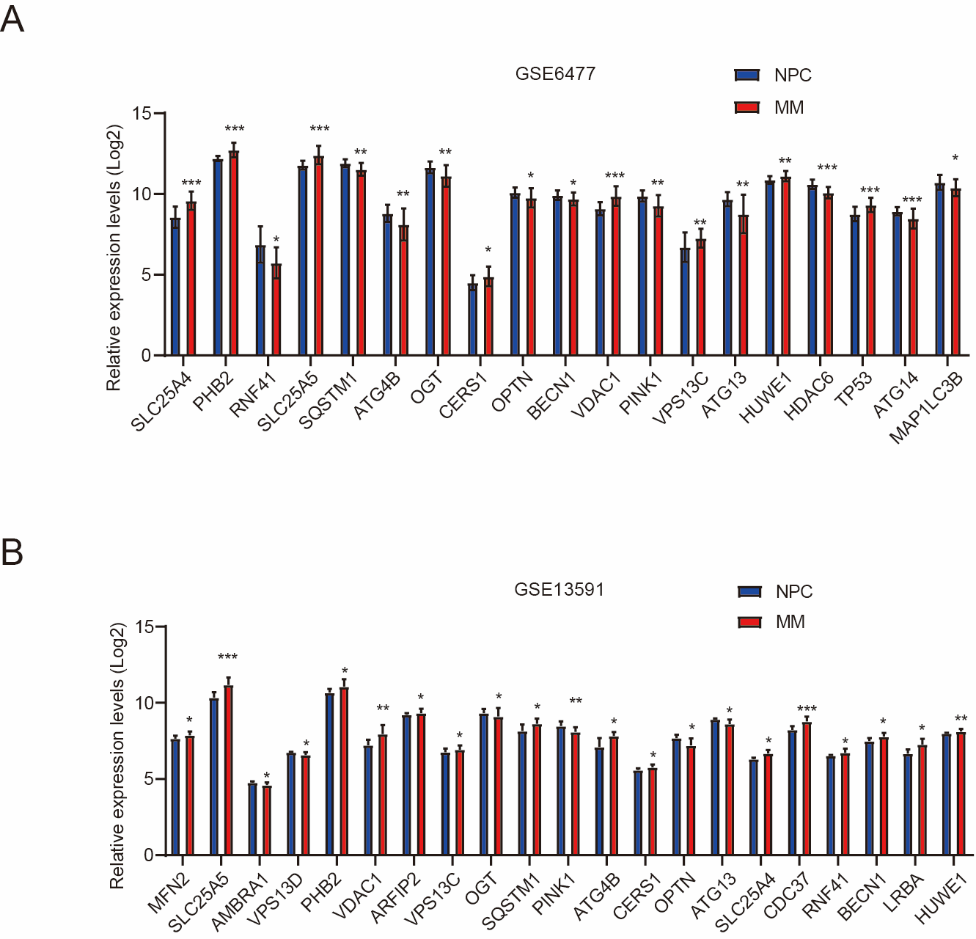
**

**Supplementary Figure 2 The expression of differentially expressed mitophagy-related genes in MM.**

(A). The bar chart showed the expression of 19 differentially expressed mitophagy-related genes in 15 NPC and 73 MM samples from the GSE6477 dataset. (B). The bar chart showed the expression of 21 differentially expressed mitophagy-related genes in 5 NPC and 133 MM samples from the GSE13591 dataset. * *p*<0.05, ** *p*<0.01, *** *p*<0.001.


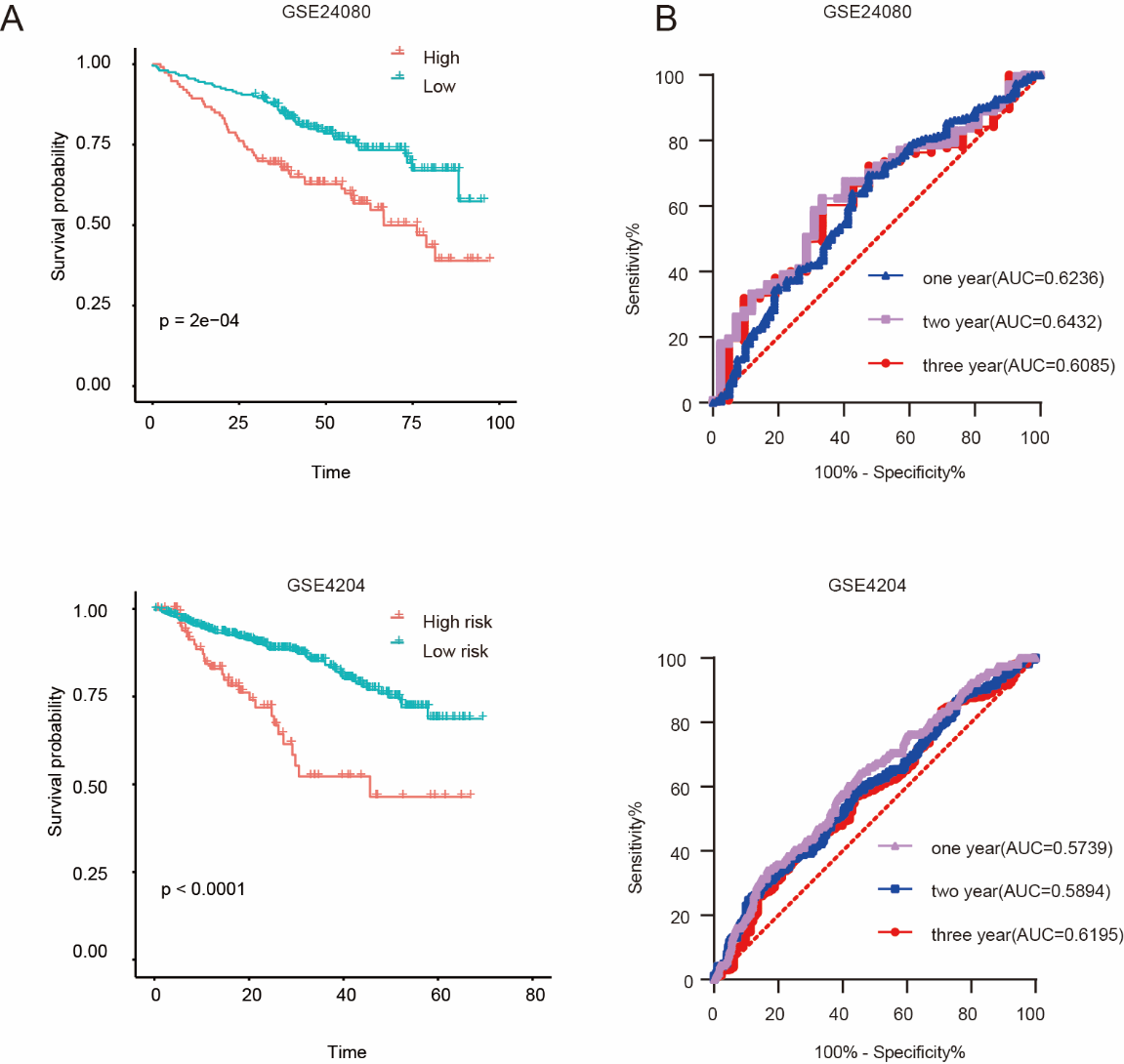


**Supplementary Figure 3 validation of prognostic risk model**

(A). Kaplan-Meier survival analysis in validation cohort GSE24080 and GSE4204 based on the risk score. (B). ROC curve for the prognostic risk signature in validation cohort GSE24080 and GSE4204.


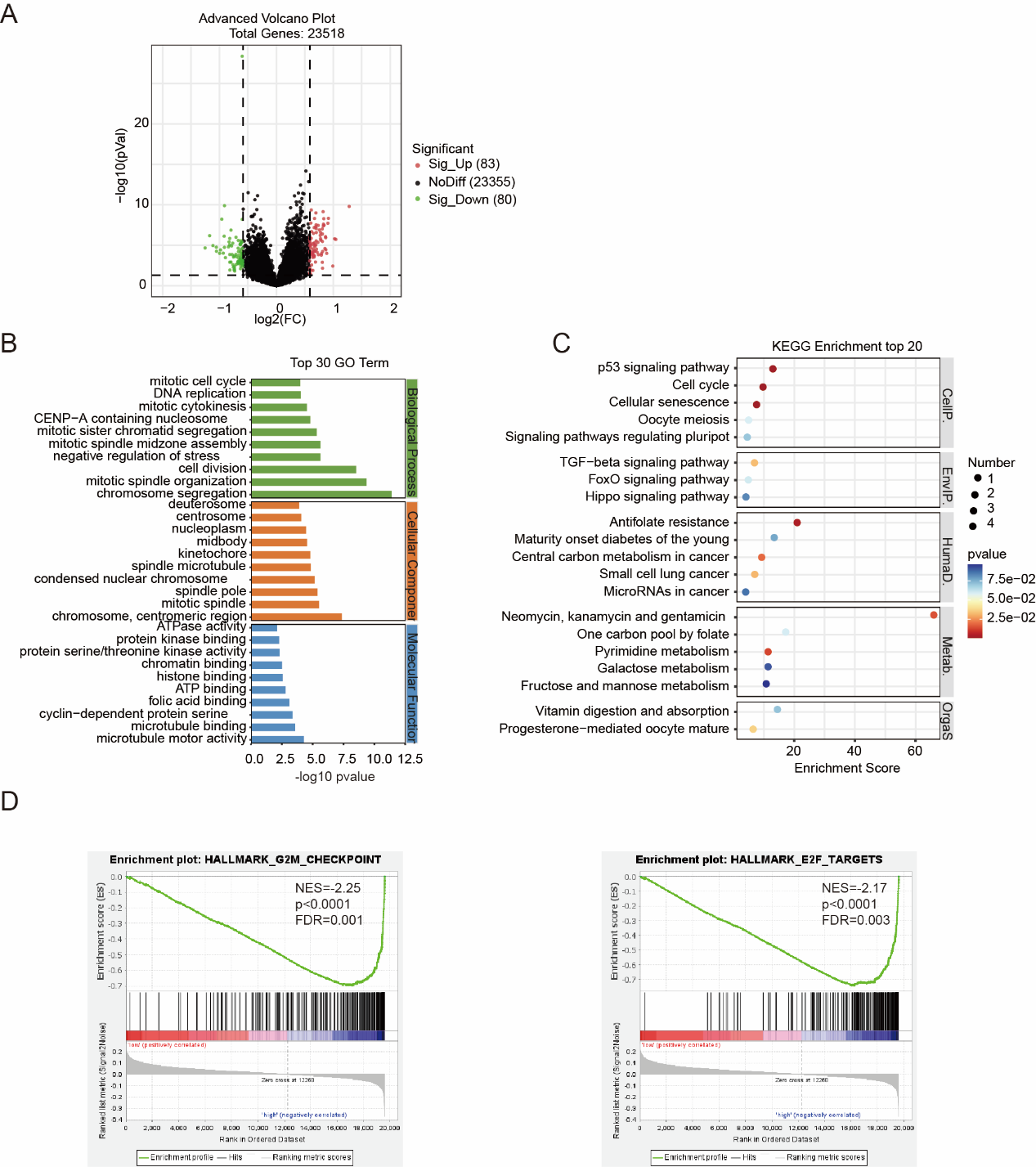


**Supplementary Figure 4. Enrichment analysis of mitophagy-related risk signature**

(A). Volcano plot for differentially expressed genes stratified by risk score. |LogFC|>0.58 and adjusted P value<0.05 was the cut-off criteria. (B). The significantly enriched gene ontology biological process of upregulated DEGs. (C). The significantly enriched KEGG pathway of upregulated DEGs. (D). GSEA analysis results of HALLMARK gene set. NES, normalized enrichment score; FDR, false discovery rate.
