# Supplementary material for: Integrative analysis of the prognostic value and immune microenvironment of mitophagy-related signature for multiple myeloma
Source: BMC Cancer. 2023 Sep 12;23:859. doi: 10.1186/s12885-023-11371-7 (PMC10496355; doi:10.1186/s12885-023-11371-7)
Supplement: Supplementary file 2 — Supplementary Material 2 [file 12885_2023_11371_MOESM2_ESM.docx]

**Supplementary Table 1. The information of gene sets used in this study.**

| Accession number | Platform | Number of samples | Country | Years | age(median) | gender(male/female) | race(white/others) | Treatment |
| --- | --- | --- | --- | --- | --- | --- | --- | --- |
| GSE6477 | GPL96 | 15NPC, 22 MGUS, 24 SMM, 73 MM and 28 RRMM | USA | 2007 | / | / | / | / |
| GSE13591 | GPL96 | 5NPC, 11 MGUS, 133 MM and 9 PCL | Italy | 2009 | / | / | / | / |
| GSE9782 | GPL96 and GPL97 | 264 MM | USA | 2007 | 61 | 159/105 | 229/35 | DEX:77  Btz: 189 |
| GSE24080 | GPL570 | 554 MM | China | 2010 | 57.75 | 195/118 | 270/43 | TT2: 351  TT3: 214 |
| GSE4204 | GPL570 | 538 MM | USA | 2006 | / | / | / | TT2:345  TT3:193 |
| GSE47552 | GPL6244 | 5NPC, 20 MGUS, 33 SMM, 41 MM | Spain | 2014 | / | / | / | / |

NPC: normal plasma cells; MGUS: monoclonal gammopathy of unknown significance; SMM: smoldering multiple myeloma; MM: multiple myeloma; RRMM: refractory and/or relapse MM; PCL: plasma cell leukemia.

**Supplementary Table 2 The mitophagy-related genes in GSEA database**

| Standard name | Systematic name | Number of gene | Related gene | Brief description |
| --- | --- | --- | --- | --- |
| GOBP_REGULATION_OF_MITOPHAGY | M14044 | 13 | AMBRA1, CERS1, PINK1, RNF41, SLC25A4, SLC25A5, TIGAR, TP53, TSC2, USP30, VDAC1, VPS13C, VPS13D | Any process that modulates the frequency, rate or extent of macromitophagy. [GOC:TermGenie]/ |
| GOBP_MITOPHAGY | M22148 | 36 | AMBRA1, ARFIP2, ATG13, ATG14, ATG4B, ATG4D, BECN1, CDC37, CERS1, HDAC6, HTRA2, HUWE1, LRBA, MAP1LC3B, MFN2, OGT, OPTN, PHB2, PINK1, PRKN, RETREG1, RIMOC1, R NF41, SLC25A4, SLC25A5, SPATA33, SQSTM1, TAFAZZIN, TIGAR, TOMM7, TP53, TSC2, USP30, VDAC1, VPS13C, VPS13D | The selective autophagy process in which a mitochondrion is degraded by macroautophagy. [PMID:15798367]/ |
| GOBP_POSITIVE_REGULATION_OF_MITOPHAGY_IN_RESPONSE_TO_MITOCHONDRIAL_DEPOLARIZATIO | M40519 | 10 | CDC37, HDAC6, HUWE1, MFN2, OPTN, PINK1, PRKN, TOMM7, VDAC1, VPS13C | Any process that activates or increases the frequency, rate or extent of mitophagy in response to mitochondrial depolarization. [GOC:PARL, PMID:18200046, PMID:23985961] |
| GOBP_POSITIVE_REGULATION_OF_MITOPHAGY | M43001 | 6 | AMBRA1, CERS1, SLC25A4, SLC25A5, VDAC1, VPS13D | Any process that activates or increases the frequency, rate or extent of mitophagy. [GOC:TermGenie] |
| GOBP_PARKIN_MEDIATED_STIMULATION_OF_MITOPHAGY_IN_RESPONSE_TO_MITOCHONDRIAL_DEPOLARIZATION | M24306 | 6 | HDAC6, MFN2, OPTN, PRKN, VDAC1, VPS13C | A positive regulation of the macromitophagy pathway that is triggered by mitochondrial depolarization and requires the function of a parkin-family molecule. [GOC:autophagy, GOC:dph, GOC:pad, GOC:PARL, PMID:25349190] |

**Supplementary Table 3 Impact of mitophagy-related genes on the most common cytogenetic abnormalities of multiple myeloma (GSE136337).**

| Gene_id | del13q  Cytogenetic  abnormalities | del11q | del17p | del16q | del1p32 | del1p | del11q | Amp1q | myc_8q24 | x1qplus | hyperdiploid | t_11_14 |
| --- | --- | --- | --- | --- | --- | --- | --- | --- | --- | --- | --- | --- |
| VDAC1 | 0.003 | 0.142 | 0.062 | 0.025 | 0.001 | <0.0001 | 0.001 | 0.482 | 0.008 | <0.0001 | <0.0001 | 0.59 |
| PINK1 | 0.001 | 0.899 | 0.704 | 0.235 | 0.006 | 0.007 | 0.009 | 0.818 | 0.014 | <0.0001 | <0.0001 | 0.876 |
| VPS13C | 0.104 | 0.413 | 0.176 | 0.911 | 0.332 | 0.294 | 0.344 | 0.272 | 0.498 | 0.813 | 0.633 | 0.341 |
| ATG13 | 0.305 | 0.118 | 0.532 | 0.44 | 0.260 | 0.413 | 0.184 | 0.017 | 0.225 | 0.544 | 0.322 | 0.139 |
| HUWE1 | 0.352 | 0.343 | 0.626 | 0.751 | 0.192 | 0.100 | 0.247 | 0.487 | 0.425 | 0.628 | 0.616 | 0.802 |

Red means upregulated, green means downregulated.

**Supplementary Table 4 Differentially expressed genes of MM patients between high- and low-risk groups**

| gene | logFC |  | AveExpr | t | P.Value | adj.P.Val | B |
| --- | --- | --- | --- | --- | --- | --- | --- |
| MATR3 | 1.279139 |  | 7.740953 | 6.616595 | 1.58E-10 | 1.95E-07 | 13.52548 |
| SLC19A1 | 0.614089 |  | 6.053927 | 6.443455 | 4.36E-10 | 4.28E-07 | 12.56579 |
| SNORD88C | 0.820782 |  | 8.691613 | 6.35967 | 7.09E-10 | 6.18E-07 | 12.10831 |
| UBE2T | 0.695012 |  | 8.658588 | 6.299154 | 1E-09 | 8.15E-07 | 11.78073 |
| RP1-130G2.1 | 0.914994 |  | 7.385759 | 6.024825 | 4.72E-09 | 2.58E-06 | 10.32621 |
| DTL | 0.671094 |  | 8.729574 | 6.018408 | 4.89E-09 | 2.61E-06 | 10.29279 |
| RRM2 | 0.834027 |  | 10.57909 | 6.008746 | 5.15E-09 | 2.69E-06 | 10.24252 |
| CKS2 | 0.605075 |  | 11.05503 | 5.780701 | 1.79E-08 | 6.88E-06 | 9.074769 |
| HPDL | 0.90498 |  | 6.389201 | 5.771273 | 1.88E-08 | 7.02E-06 | 9.027264 |
| TACC3 | 0.82744 |  | 6.046217 | 5.716893 | 2.52E-08 | 8.96E-06 | 8.754493 |
| SPC25 | 0.69895 |  | 7.320255 | 5.685421 | 2.97E-08 | 1.03E-05 | 8.597574 |
| MCM10 | 0.839457 |  | 6.548132 | 5.666183 | 3.29E-08 | 1.11E-05 | 8.502002 |
| KIF23 | 0.734826 |  | 6.023184 | 5.652563 | 3.54E-08 | 1.17E-05 | 8.434492 |
| DEPDC1B | 0.823582 |  | 7.417328 | 5.622265 | 4.15E-08 | 1.32E-05 | 8.284797 |
| TRIP13 | 0.690497 |  | 7.967089 | 5.56688 | 5.55E-08 | 1.65E-05 | 8.012838 |
| CDK1 | 0.663397 |  | 8.346856 | 5.534253 | 6.58E-08 | 1.84E-05 | 7.853651 |
| FAM72A | 0.700542 |  | 8.734666 | 5.480076 | 8.71E-08 | 2.25E-05 | 7.591021 |
| HJURP | 0.808331 |  | 7.889493 | 5.462369 | 9.54E-08 | 2.39E-05 | 7.505641 |
| CENPK | 0.756158 |  | 8.168557 | 5.407784 | 1.26E-07 | 3E-05 | 7.243871 |
| CCDC78 | 0.619843 |  | 7.398317 | 5.309116 | 2.08E-07 | 4.37E-05 | 6.776206 |
| KIF4A | 0.778075 |  | 7.338025 | 5.262993 | 2.62E-07 | 5.28E-05 | 6.560048 |
| TTK | 0.701966 |  | 7.794697 | 5.258613 | 2.68E-07 | 5.3E-05 | 6.539601 |
| OIP5 | 0.652262 |  | 7.926668 | 5.206672 | 3.47E-07 | 6.59E-05 | 6.298222 |
| RRN3P2 | 0.810846 |  | 6.435299 | 5.175687 | 4.05E-07 | 7.43E-05 | 6.155181 |
| KIF20A | 0.77055 |  | 7.548545 | 5.091848 | 6.11E-07 | 9.98E-05 | 5.771739 |
| MYC | 0.865162 |  | 12.20616 | 5.017992 | 8.74E-07 | 0.000125 | 5.438329 |
| TYMS | 0.590943 |  | 9.173494 | 4.973744 | 1.08E-06 | 0.000144 | 5.240558 |
| FOXM1 | 0.868157 |  | 5.266464 | 4.95599 | 1.18E-06 | 0.000155 | 5.161622 |
| PBK | 0.611446 |  | 8.136391 | 4.921373 | 1.39E-06 | 0.000176 | 5.008399 |
| CENPA | 0.775935 |  | 7.762274 | 4.899049 | 1.54E-06 | 0.000189 | 4.910077 |
| BC017398 | 1.024173 |  | 8.398454 | 4.89427 | 1.58E-06 | 0.000191 | 4.889074 |
| HK2 | 1.044937 |  | 8.942767 | 4.85461 | 1.9E-06 | 0.000219 | 4.715481 |
| NEK2 | 0.587277 |  | 7.623865 | 4.815706 | 2.28E-06 | 0.000251 | 4.546372 |
| NDC80 | 0.782193 |  | 7.753844 | 4.811168 | 2.33E-06 | 0.000254 | 4.526724 |
| UHRF1 | 0.699881 |  | 8.287061 | 4.75822 | 2.98E-06 | 0.000301 | 4.298631 |
| SKA1 | 0.810367 |  | 6.038096 | 4.726107 | 3.45E-06 | 0.000334 | 4.161355 |
| PRC1 | 0.5894 |  | 9.326313 | 4.710769 | 3.71E-06 | 0.000351 | 4.096069 |
| RP11-480A16.1 | 0.731308 |  | 7.274219 | 4.674404 | 4.37E-06 | 0.000388 | 3.942017 |
| FCHO1 | 0.668457 |  | 7.030745 | 4.63182 | 5.31E-06 | 0.000446 | 3.762933 |
| PLK4 | 0.647383 |  | 5.838674 | 4.613237 | 5.77E-06 | 0.000471 | 3.685231 |
| CCNB1 | 0.601289 |  | 7.842926 | 4.57076 | 6.99E-06 | 0.000548 | 3.508639 |
| DUXAP10 | 0.694915 |  | 7.84882 | 4.525768 | 8.54E-06 | 0.000635 | 3.323143 |
| CKAP2L | 0.606345 |  | 7.362068 | 4.490172 | 9.99E-06 | 0.000706 | 3.177519 |
| TRMT1 | 0.664269 |  | 8.482978 | 4.480221 | 1.04E-05 | 0.000722 | 3.136988 |
| RP11-66N11.7 | 0.58606 |  | 6.051588 | 4.464255 | 1.12E-05 | 0.000753 | 3.072126 |
| SSX4 /// SSX4B | 0.680238 |  | 7.74563 | 4.426686 | 1.32E-05 | 0.000844 | 2.920301 |
| MNX1 | 0.603483 |  | 5.568804 | 4.303503 | 2.25E-05 | 0.001249 | 2.430424 |
| DUXAP10 | 0.853202 |  | 5.666898 | 4.270492 | 2.58E-05 | 0.001394 | 2.301222 |
| HIST1H3C | 0.651639 |  | 4.713436 | 4.250161 | 2.82E-05 | 0.001479 | 2.222089 |
| NUF2 | 0.70518 |  | 7.527177 | 4.232457 | 3.03E-05 | 0.001555 | 2.153452 |
| MAGEA6 | 0.869749 |  | 8.428916 | 4.18879 | 3.64E-05 | 0.001767 | 1.985249 |
| PCDHB2 | 0.678379 |  | 5.86807 | 4.097758 | 5.31E-05 | 0.00232 | 1.639603 |
| POLE2 | 0.640862 |  | 7.073103 | 4.093007 | 5.41E-05 | 0.002345 | 1.62175 |
| ZDHHC12 | 0.58642 |  | 8.111472 | 4.090739 | 5.46E-05 | 0.002354 | 1.613235 |
| LINC00403 | 0.813293 |  | 4.129332 | 4.07521 | 5.82E-05 | 0.002463 | 1.555038 |
| ZNF569 | 0.668382 |  | 5.946633 | 4.07082 | 5.93E-05 | 0.002503 | 1.538621 |
| XAGE1B | 0.715302 |  | 6.239045 | 4.02284 | 7.2E-05 | 0.002866 | 1.360243 |
| SNORA5B | 0.622686 |  | 7.799368 | 3.982755 | 8.46E-05 | 0.003226 | 1.212676 |
| PRR3 | 0.593143 |  | 7.272372 | 3.973349 | 8.79E-05 | 0.003291 | 1.178243 |
| KIF18A | 0.636992 |  | 6.979846 | 3.971586 | 8.85E-05 | 0.003309 | 1.171797 |
| MAGEA3 | 0.885117 |  | 8.633253 | 3.936338 | 0.000102 | 0.003595 | 1.043461 |
| TOP2A | 0.793214 |  | 7.485898 | 3.926119 | 0.000106 | 0.003684 | 1.006448 |
| NCAPH | 0.630695 |  | 5.940642 | 3.8721 | 0.000131 | 0.004262 | 0.812236 |
| LINC01021 | 0.738741 |  | 6.016717 | 3.842249 | 0.000147 | 0.004598 | 0.70596 |
| ARHGAP11B | 0.648872 |  | 6.12841 | 3.835424 | 0.000151 | 0.004678 | 0.681768 |
| EMC3-AS1 | 0.622891 |  | 5.998597 | 3.829227 | 0.000155 | 0.00478 | 0.659835 |
| LOC283352 | 0.789724 |  | 7.440602 | 3.807873 | 0.000168 | 0.005092 | 0.5845 |
| F2R | 0.665931 |  | 8.017352 | 3.76347 | 0.0002 | 0.005835 | 0.429083 |
| AC004941.5 | 0.661236 |  | 6.406078 | 3.760719 | 0.000202 | 0.005877 | 0.419506 |
| LOC100101148 | 0.661279 |  | 6.987717 | 3.659735 | 0.000296 | 0.007706 | 0.072474 |
| FABP5 | 0.63481 |  | 9.309915 | 3.649655 | 0.000307 | 0.007906 | 0.038307 |
| C1orf112 | 0.585654 |  | 6.164391 | 3.357838 | 0.000882 | 0.016724 | -0.91313 |
| GAGE12B | 0.686703 |  | 5.387662 | 3.269898 | 0.001195 | 0.020216 | -1.18544 |
| SMAD1 | 0.808735 |  | 9.654015 | 3.246008 | 0.001296 | 0.021306 | -1.25826 |
| LOC101928433 | 0.620946 |  | 6.040142 | 3.021711 | 0.002719 | 0.035413 | -1.91748 |
| GAGE1 | 0.580026 |  | 6.766707 | 2.938362 | 0.003543 | 0.042378 | -2.15113 |
| LAMP5 | 0.991374 |  | 11.38206 | 2.91816 | 0.003774 | 0.043811 | -2.20684 |
| DUSP2 | 0.589063 |  | 7.931469 | 2.721214 | 0.006865 | 0.064151 | -2.73077 |
| SCN3A | 0.811918 |  | 7.160753 | 2.713055 | 0.007033 | 0.065119 | -2.75173 |
| NRN1 | 0.599182 |  | 7.616918 | 2.547763 | 0.011316 | 0.087992 | -3.1633 |
| TRAT1 | 0.61154 |  | 8.875683 | 2.497672 | 0.01301 | 0.095831 | -3.28314 |
| PTPN20B | 0.642361 |  | 4.839966 | 2.494788 | 0.013114 | 0.096507 | -3.28997 |
| LHX8 | 0.586646 |  | 5.351609 | 2.198333 | 0.028651 | 0.155768 | -3.95144 |
| TSPY1 | 0.59776 |  | 4.664047 | 2.121171 | 0.034687 | 0.174206 | -4.11035 |
| PINK1 | -0.60037 |  | 10.20942 | -12.4185 | 4.24E-29 | 4.98E-25 | 54.02557 |
| GABARAPL1 | -0.91151 |  | 9.984485 | -6.64971 | 1.29E-10 | 1.69E-07 | 13.71117 |
| SLC7A7 | -0.5826 |  | 10.72375 | -6.48712 | 3.38E-10 | 3.62E-07 | 12.806 |
| CD27 | -0.95987 |  | 11.12485 | -5.97978 | 6.05E-09 | 3.03E-06 | 10.09221 |
| SELM | -0.59866 |  | 12.14623 | -5.97024 | 6.38E-09 | 3.12E-06 | 10.04283 |
| RAP1GAP2 | -0.79781 |  | 8.637377 | -5.39485 | 1.35E-07 | 3.14E-05 | 7.182142 |
| IGKV1OR2-108 | -1.17328 |  | 12.24566 | -5.07978 | 6.48E-07 | 0.000102 | 5.716968 |
| IGK /// IGKC | -0.91702 |  | 12.89681 | -5.04537 | 7.66E-07 | 0.000113 | 5.561442 |
| LOC100507562 | -0.70901 |  | 7.177521 | -4.98589 | 1.02E-06 | 0.000139 | 5.294686 |
| PCDHAC1 | -0.81933 |  | 8.327683 | -4.91531 | 1.43E-06 | 0.000177 | 4.981648 |
| LRRK2 | -0.66908 |  | 8.962778 | -4.79257 | 2.54E-06 | 0.000272 | 4.446341 |
| RAB3B | -0.62026 |  | 7.308235 | -4.76894 | 2.83E-06 | 0.000294 | 4.344616 |
| LOC100131662 | -0.83093 |  | 6.353681 | -4.70397 | 3.82E-06 | 0.000356 | 4.067198 |
| ALDH2 | -0.60496 |  | 11.35903 | -4.69181 | 4.04E-06 | 0.000367 | 4.015628 |
| TTLL7 | -0.61543 |  | 7.869771 | -4.63522 | 5.23E-06 | 0.000442 | 3.777162 |
| APOC1 | -0.76688 |  | 7.675555 | -4.62239 | 5.54E-06 | 0.00046 | 3.723458 |
| PCOLCE-AS1 | -0.63133 |  | 8.958111 | -4.59655 | 6.22E-06 | 0.000499 | 3.615692 |
| MVD | -0.63846 |  | 6.574902 | -4.54507 | 7.83E-06 | 0.000601 | 3.402506 |
| ZNF549 | -0.63043 |  | 6.210632 | -4.52023 | 8.75E-06 | 0.000649 | 3.300402 |
| Igk | -0.82159 |  | 8.577803 | -4.51197 | 9.08E-06 | 0.000671 | 3.266554 |
| IGKV1-37 | -1.11396 |  | 11.81442 | -4.46232 | 1.13E-05 | 0.000757 | 3.064272 |
| AP001605.4 | -0.78617 |  | 6.969954 | -4.44371 | 1.23E-05 | 0.000798 | 2.98897 |
| SNX9 | -0.84085 |  | 7.551337 | -4.38059 | 1.61E-05 | 0.000983 | 2.735564 |
| IGHV3-73 | -1.05394 |  | 5.542886 | -4.35765 | 1.78E-05 | 0.001058 | 2.644252 |
| AC128677.4 | -0.91282 |  | 12.53467 | -4.35055 | 1.84E-05 | 0.001077 | 2.616099 |
| abParts /// IGKC | -1.25229 |  | 11.19121 | -4.31511 | 2.14E-05 | 0.001199 | 2.47607 |
| MAST1 | -0.67097 |  | 6.409096 | -4.29803 | 2.30E-05 | 0.001269 | 2.40895 |
| HLA-DQB1 | -0.59894 |  | 9.905819 | -4.29612 | 2.32E-05 | 0.001271 | 2.401447 |
| MARCKS | -0.62308 |  | 10.69262 | -4.18992 | 3.63E-05 | 0.001766 | 1.989567 |
| IGKV1-17 | -0.98157 |  | 12.40694 | -4.17685 | 3.83E-05 | 0.001823 | 1.939521 |
| IGHD | -1.0515 |  | 7.620591 | -4.08682 | 5.55E-05 | 0.002379 | 1.598537 |
| NR3C2 | -0.66661 |  | 7.270639 | -4.06269 | 6.13E-05 | 0.002554 | 1.508278 |
| CD74 | -0.63026 |  | 10.73656 | -4.01873 | 7.32E-05 | 0.002895 | 1.345069 |
| HLA-DRA | -0.93381 |  | 8.886713 | -3.95039 | 9.63E-05 | 0.003479 | 1.094509 |
| SLC46A3 | -0.60594 |  | 8.801031 | -3.93804 | 0.000101 | 0.003589 | 1.049626 |
| IGHA1 | -0.99644 |  | 7.2835 | -3.93799 | 0.000101 | 0.003589 | 1.04944 |
| HLA-DRB1 | -0.5858 |  | 9.487485 | -3.92699 | 0.000106 | 0.003677 | 1.00961 |
| PLA2G7 | -0.7109 |  | 6.198399 | -3.85895 | 0.000138 | 0.004425 | 0.765322 |
| IGKC | -0.8186 |  | 11.66653 | -3.85315 | 0.000141 | 0.004472 | 0.74467 |
| S100A8 | -0.77804 |  | 7.901788 | -3.85157 | 0.000142 | 0.004494 | 0.739062 |
| DEFA1 | -0.60504 |  | 8.847948 | -3.84366 | 0.000147 | 0.004585 | 0.71096 |
| IGH | -0.65688 |  | 13.93842 | -3.79417 | 0.000178 | 0.005301 | 0.536354 |
| LOC100293211 | -0.7387 |  | 8.032 | -3.74366 | 0.000215 | 0.006155 | 0.360264 |
| SCNN1B | -0.60154 |  | 8.431887 | -3.74159 | 0.000217 | 0.006167 | 0.353103 |
| CKAP2 | -0.69752 |  | 8.476639 | -3.67595 | 0.000278 | 0.007392 | 0.127604 |
| IGLJ3 | -0.75231 |  | 9.648714 | -3.66712 | 0.000288 | 0.007574 | 0.097569 |
| PTGER2 | -0.636 |  | 5.914267 | -3.62374 | 0.000338 | 0.008439 | -0.04914 |
| S100A12 | -0.89418 |  | 7.788271 | -3.61848 | 0.000345 | 0.008577 | -0.0668 |
| LRP11 | -0.6079 |  | 8.117768 | -3.54961 | 0.000445 | 0.01031 | -0.29609 |
| MAGED4 | -0.65812 |  | 7.675244 | -3.54472 | 0.000453 | 0.010455 | -0.31223 |
| MDK | -0.60067 |  | 8.950976 | -3.52862 | 0.00048 | 0.010872 | -0.36519 |
| ELANE | -0.59744 |  | 5.837843 | -3.51126 | 0.000511 | 0.011383 | -0.42202 |
| P2RX1 | -0.63351 |  | 10.24124 | -3.50413 | 0.000524 | 0.011602 | -0.44529 |
| IFI6 | -0.58189 |  | 10.45307 | -3.42943 | 0.000685 | 0.014003 | -0.68651 |
| FBP1 | -0.63002 |  | 9.849197 | -3.37003 | 0.000845 | 0.016361 | -0.87485 |
| TNFSF8 | -0.59023 |  | 7.563458 | -3.36523 | 0.000859 | 0.016511 | -0.88993 |
| IGHV3-54 | -0.60197 |  | 8.820193 | -3.36279 | 0.000867 | 0.016584 | -0.8976 |
| VCAM1 | -0.67482 |  | 9.138248 | -3.31621 | 0.001019 | 0.018378 | -1.04289 |
| CKAP2 | -0.58433 |  | 10.78226 | -3.31066 | 0.001039 | 0.018576 | -1.06006 |
| CPVL | -0.66959 |  | 8.821765 | -3.30259 | 0.001068 | 0.018928 | -1.08498 |
| ISLR | -0.63005 |  | 7.54185 | -3.29718 | 0.001088 | 0.01914 | -1.10167 |
| C1QA | -0.79803 |  | 7.571918 | -3.26847 | 0.001201 | 0.020286 | -1.18982 |
| S100A9 | -0.81839 |  | 7.596951 | -3.25451 | 0.001259 | 0.020826 | -1.23239 |
| ENO2 | -0.65998 |  | 9.353625 | -3.23945 | 0.001325 | 0.021661 | -1.27816 |
| HMOX1 | -0.60291 |  | 9.15464 | -3.21975 | 0.001417 | 0.022576 | -1.33772 |
| SPINT2 | -0.6395 |  | 10.49033 | -3.19996 | 0.001514 | 0.023644 | -1.39719 |
| IGHG1 /// IGHM | -0.67653 |  | 7.706875 | -3.17504 | 0.001646 | 0.025074 | -1.47161 |
| IGKC /// IGKV1-5 /// IGKV1-5 /// | -0.61845 |  | 6.13142 | -3.16298 | 0.001714 | 0.025638 | -1.50745 |
| IGHA1 /// IGHG1 /// IGHM /// IGHV3-23 /// IGHV4-31 | -0.5912 |  | 8.683879 | -3.16195 | 0.001719 | 0.025676 | -1.51047 |
| IGHA1 /// IGHG1 /// IGHM | -0.65915 |  | 8.8284 | -3.12743 | 0.001928 | 0.027938 | -1.61227 |
| A1BG | -0.60991 |  | 7.204985 | -3.11003 | 0.002041 | 0.029081 | -1.66319 |
| CD99 | -0.59324 |  | 9.153651 | -3.09706 | 0.00213 | 0.029874 | -1.70097 |
| Ig alpha 1-[alpha]2m /// IGH | -0.70323 |  | 9.805181 | -3.06816 | 0.002341 | 0.031709 | -1.78459 |
| CD163 | -0.76261 |  | 7.586511 | -3.04399 | 0.002531 | 0.03369 | -1.85398 |
| IGHA1 /// IGHA2 /// IGHG1 /// IGHG3 /// IGHM /// IGHV3-23 /// IGHV4-31 | -0.6227 |  | 11.03307 | -2.91652 | 0.003793 | 0.043971 | -2.21135 |
| IGHA1 /// IGHA2 /// IGHD /// IGHG1 /// IGHG3 /// IGHM /// IGHV4-31 | -0.59027 |  | 11.10924 | -2.8851 | 0.004182 | 0.046796 | -2.2972 |
| IGHA1 /// IGHA2 /// IGHD /// IGHG1 /// IGHG3 /// IGHG4 /// IGHM /// IGHV4-31 | -0.73446 |  | 9.808205 | -2.87544 | 0.004309 | 0.047544 | -2.32343 |
| IGH /// IGHA1 /// IGHD /// IGHG1 /// IGHG3 /// IGHM /// IGHV3-23 /// IGHV4-31 | -0.7548 |  | 9.96654 | -2.81461 | 0.005191 | 0.05359 | -2.48664 |
| CTHRC1 | -0.63012 |  | 11.18192 | -2.66457 | 0.008105 | 0.071162 | -2.87502 |
| CTSW | -0.73454 |  | 6.954734 | -2.64432 | 0.008595 | 0.073641 | -2.92588 |
| SLC2A10 | -0.74312 |  | 6.028587 | -2.55549 | 0.011073 | 0.08701 | -3.14462 |
| IGLC1 | -0.81664 |  | 9.459629 | -2.53385 | 0.011765 | 0.090251 | -3.19681 |
| HBA1 /// HBA2 | -0.73167 |  | 9.987445 | -2.43574 | 0.015415 | 0.10607 | -3.42815 |
| RGS13 | -0.5803 |  | 6.458937 | -1.98524 | 0.047984 | 0.210469 | -4.37687 |

**Supplementary Table 5 Primers for qRT-PCR used in this study.**

| **gene** | **Forward primer** | **Reverse primer** |
| --- | --- | --- |
| VDAC1 | ACGTATGCCGATCTTGGCAAA | TCAGGCCGTACTCAGTCCATC |
| PINK1 | GCCTCATCGAGGAAAAACAGG | GTCTCGTGTCCAACGGGTC |
| VPS13C | TGTGGAAAAATTGGCAACTCAAG | CCCAGTGTGACACCAAATGAA |
| ATG13 | TTGCTATAACTAGGGTGACACCA | CCCAACACGAACTGTCTGGA |
| HUWE1 | TGCCAGTGCTTGTAAGGAACT | TGGTGACAAATGTTATCTGGTCC |
| GAPDH | CGGAGTCAACGGATTTGGTCGTAT | AGCCTTCTCCATGGTGGTGAAGAC |
