# Supplementary material for: Integrative analysis of the prognostic value and immune microenvironment of mitophagy-related signature for multiple myeloma
Source: BMC Cancer. 2023 Sep 12;23:859. doi: 10.1186/s12885-023-11371-7 (PMC10496355; doi:10.1186/s12885-023-11371-7)
Supplement: Supplementary file 3 — Supplementary Material 3 [file 12885_2023_11371_MOESM3_ESM.docx]

Supplementary Material

**Methods**

**Verification of differentially expressed mitophagy-related genes**

We verified differentially expressed mitophagy-related genes in GSE6477 and GSE13591 by “limma” R package, with p value<0.05. The DEGs upon risk score in GSE24080 were identified by |Log_2_FC|>0.58 and adjusted *p* value<0.05. The “heatmap” R package was used for visualization.

**Protein-Protein interaction (PPI) network and Spearman’s correlation**

We used the STRING online database (https://cn.string-db.org/) to build the PPI network upon the 15 differentially expressed mitophagy-related genes. Cytoscape software (version 3.8.2) was applied to showing details [1]. The relationship among 15 mitophagy-related genes was demonstrated by Spearman’s correlation coefficient. The “corrgram” R package (version 1.14) was utilized for visualization.

**Establishment of the prognostic risk model**

First, univariate cox regression analysis was applied to obtain the OS-related genes with p<0.05, and ten genes (SLC25A4, VDAC1, RNF41, SLC25A5, PINK1, SQSTM1, VPS13C, ATG13, HUWE1, and OPTN) were significantly correlated with MM OS time. Next, we performed LASSO Cox regression to optimize the prognostic model by further compressing the genes and constructing the prognostic model by “glmnet” package (version 4.1-1), and five genes finally came into the risk score formula (VDAC1, PINK1, VPS13C, ATG13, and HUWE1). Moreover, MM patients were divided into two groups upon the optimal cutoff of the risk score with “Survminer” package (version 0.4.9). Receiver operating characteristic (ROC) curve was applied to estimate the prognostic value of risk score model in MM patients.

**Construction of the nomogram**

To assess the prediction value of risk score in MM, we performed univariate and multivariate cox regression analysis. Variables with p<0.10 was supposed to the multivariate cox regression analysis, and p<0.05 was considered as remarkable independent prognostic factors. Then, we used “rms” package (version 6.2-0) to construct the nomogram independent prognostic model. Finally, we assessed the predictive value of nomogram by ROC curve and calibration curve.

**Functional pathway enrichment analysis**

We used the online website Metascape (http://metascape.org/gp/index.html) to analyze gene ontology (GO) and Kyoto Encyclopedia of Genes and Genomes (KEGG) pathway [2-4] of DEGs based on risk score group. We executed Gene set enrichment analysis (GSEA) by GSEA software (version 4.1.0). The absolute value of NES value>1, p value <0.05, and false discovery rate (FDR) value <0.25 showed significance.

**Estimation of immune infiltration**

We compute d the immune score, stromal score, tumor purity, and ESTIMATE score for each sample in GSE24080 by ESTIMATE algorithm [5]. According to the previous study [6], single sample GSEA (ssGSEA) was conducted to evaluate the abundance of infiltrating cells by “GSVA” package (version 1.38.2).

**Prediction of drug sensitivity**

To achieve precise treatment upon mitophagy-related signature and identified potential drugs for MM, we utilized the Genomics of drugs sensitivity in cancer (GDSC, https://www.cancerrxgene.org/) to predict the chemotherapeutic response [7]. R package “pRROphetic” was implemented to evaluate the half-maximal inhibitory concentration (IC50) by ridge regression [8]. The forecast precision was determined through 10-fold cross-validation using the GDSC training set.

**RNA extraction and quantitative** **real-time polymerase chain reaction (qRT-PCR)**

All samples from 16 healthy donors and 16 newly diagnosed MM (NDMM) patients were purified from bone marrow aspirates from 2021-2022, which was approved by the Ethics Committee of the second affiliated hospital of Xi’an Jiaotong University, Shaanxi, China (#2022128). The written informed consent was acquired from every patient. Total RNA was extracted from the bone marrow mononuclear cells (BMMC) using TRIzol® Reagent (Invitrogen, Germany). Then NanoDrop ND-1000 was used to determine the RNA purity and concentration. The cDNA was synthesized from total RNA using a Primescript RT reagent kit (Takara). Then, the qRT-PCR was executed by SYBR Premix Ex Taq™ II (CWBIO, China). The reaction conditions were as follows: 95 °C for 30 s, 40 cycles of 95 °C for 5 s, and 60 °C for 30 s. The above experimental steps were operated in accordance with manufacturer's protocols. Primers were synthesized by Tsingke (Beijing, China) and the primer sequences were listed in Supplementary Table 5. 2^-ΔCT^ value was used to calculate the gene expression level.

**References**

1. Shannon P, Markiel A, Ozier O, Baliga NS, Wang JT, Ramage D, Amin N, Schwikowski B, Ideker T: **Cytoscape: A software environment for integrated models of biomolecular interaction networks**. *Genome Research* 2003, **13**(11):2498-2504.

2. Kanehisa M, Furumichi M, Sato Y, Kawashima M, Ishiguro-Watanabe M: **KEGG for taxonomy-based analysis of pathways and genomes**. *Nucleic Acids Res* 2023, **51**(D1):D587-D592.

3. Kanehisa M: **Toward understanding the origin and evolution of cellular organisms**. *Protein Sci* 2019, **28**(11):1947-1951.

4. Kanehisa M, Goto S: **KEGG: Kyoto Encyclopedia of Genes and Genomes**. *Nucleic Acids Res* 2000, **28**(1):27-30.

5. Yoshihara K, Shahmoradgoli M, Martínez E, Vegesna R, Kim H, Torres-Garcia W, Treviño V, Shen H, Laird PW, Levine DA *et al*: **Inferring tumour purity and stromal and immune cell admixture from expression data**. *Nat Commun* 2013, **4**:2612.

6. Jia QZ, Wu W, Wang YQ, Alexander PB, Sun CD, Gong ZH, Cheng JN, Sun HB, Guan YF, Xia XF *et al*: **Local mutational diversity drives intratumoral immune heterogeneity in non-small cell lung cancer**. *Nat Commun* 2018, **9**.

7. Lu XF, Jiang LY, Mang LY, Zhu Y, Hu WJ, Wang JS, Ruan XJ, Xu ZB, Meng XW, Gao J *et al*: **Immune Signature-Based Subtypes of Cervical Squamous Cell Carcinoma Tightly Associated with Human Papillomavirus Type 16 Expression, Molecular Features, and Clinical Outcome**. *Neoplasia* 2019, **21**(6):591-601.

8. Geeleher P, Cox N, Huang RS: **pRRophetic: an R package for prediction of clinical chemotherapeutic response from tumor gene expression levels**. (1932-6203 (Electronic)).
